# Interactome and Ubiquitinome Analyses Identify Functional Targets of Herpes Simplex Virus 1 Infected Cell Protein 0

**DOI:** 10.3389/fmicb.2022.856471

**Published:** 2022-04-18

**Authors:** Fujun Hou, Zeyu Sun, Yue Deng, Siyu Chen, Xiyuan Yang, Feiyang Ji, Menghao Zhou, Keyi Ren, Dongli Pan

**Affiliations:** ^1^State Key Laboratory for Diagnosis and Treatment of Infectious Diseases, The First Affiliated Hospital, Zhejiang University School of Medicine, Hangzhou, China; ^2^Department of Medical Microbiology and Parasitology, Zhejiang University School of Medicine, Hangzhou, China; ^3^Jinan Microecological Biomedicine Shandong Laboratory, Jinan, China

**Keywords:** herpes simplex virus, ICP0, interactome, ubiquitinome, proteomics

## Abstract

Herpes simplex virus 1 (HSV-1) can productively infect multiple cell types and establish latent infection in neurons. Infected cell protein 0 (ICP0) is an HSV-1 E3 ubiquitin ligase crucial for productive infection and reactivation from latency. However, our knowledge about its targets especially in neuronal cells is limited. We confirmed that, like in non-neuronal cells, ICP0-null virus exhibited major replication defects in primary mouse neurons and Neuro-2a cells. We identified many ICP0-interacting proteins in Neuro-2a cells, 293T cells, and human foreskin fibroblasts by mass spectrometry–based interactome analysis. Co-immunoprecipitation assays validated ICP0 interactions with acyl-coenzyme A thioesterase 8 (ACOT8), complement C1q binding protein (C1QBP), ovarian tumour domain-containing protein 4 (OTUD4), sorting nexin 9 (SNX9), and vimentin (VIM) in both Neuro-2a and 293T cells. Overexpression and knockdown experiments showed that SNX9 restricted replication of an ICP0-null but not wild-type virus in Neuro-2a cells. Ubiquitinome analysis by immunoprecipitating the trypsin-digested ubiquitin reminant followed by mass spectrometry identified numerous candidate ubiquitination substrates of ICP0 in infected Neuro-2a cells, among which OTUD4 and VIM were novel substrates confirmed to be ubiquitinated by transfected ICP0 in Neuro-2a cells despite no evidence of their degradation by ICP0. Expression of OTUD4 was induced independently of ICP0 during HSV-1 infection. Overexpressed OTUD4 enhanced type I interferon expression during infection with the ICP0-null but not wild-type virus. In summary, by combining two proteomic approaches followed by confirmatory and functional experiments, we identified and validated multiple novel targets of ICP0 and revealed potential restrictive activities of SNX9 and OTUD4 in neuronal cells.

## Introduction

Herpes simplex virus 1 (HSV-1) is a DNA virus that widely infects humans and can cause lesions in orolabial mucosa, eyes, and genital areas during productive (lytic) infection. HSV-1 gene expression during lytic infection proceeds in a cascade fashion, with immediate-early, early, and late genes being sequentially expressed. After lytic infection, HSV-1 establishes latent infection in sensory ganglia characterized by existence of viral genomes in neuronal nuclei with little protein expression but high expression of latency-associated transcripts. Latent virus can reactivate under certain stress to cause recurrent disease. This switch between lytic and latent infection enables the virus to permanently reside within the host while maintaining the ability to spread (Knipe and Howley, 2013). In rare incidences, the virus can travel from the peripheral nervous system to the central nervous system to cause life-threatening encephalitis. HSV-1 neuronal infection might also be related to chronic neurological disorders such as Alzheimer’s disease (Duarte et al., 2019).

Herpes simplex virus 1 has evolved various strategies to evade the host’s defense mechanisms. One such strategy entails encoding infected cell protein 0 (ICP0), an immediate-early protein with E3 ubiquitin ligase activity. ICP0 functions *via* its really interesting new gene (RING) finger domain to ubiquitinate its substrates for proteosomal degradation. ICP0 is also a small ubiquitin-related modifier (SUMO)–targeted ubiquitin ligase, although not all its activity depends on SUMO modification (Boutell et al., 2011; Jan Fada et al., 2020). ICP0 substrates have been reported to be involved in a wide range of host processes including intrinsic and innate immunity, chromatin remodeling, and DNA damage response (DDR) pathways (Boutell and Everett, 2003; Lilley et al., 2010; Zhang et al., 2013; Taylor and Mossman, 2015; reviewed in Lanfranca et al., 2014; Gu, 2016; Rodriguez et al., 2020). For example, ICP0-induced degradation of PML leads to the disruption of PML nuclear bodies and release of entrapped viral genomes (Everett et al., 1998, 2009, 2014; Boutell et al., 2011; Zheng et al., 2016; Dembowski and Deluca, 2017; Alandijany et al., 2018). ICP0 induces the degradation of chaperone proteins of histone H3.3 including interferon gamma inducible protein 16 (IFI16) and alpha-thalassemia/mental retardation syndrome X-linked (ATRX), resulting in attenuation of epigenetic silencing of viral genes (Jurak et al., 2012; Orzalli et al., 2012, 2015; Cuchet-Lourenco et al., 2013). ICP0 can also bind to corepressor for the re1-silencing transcription factor (CoREST) and block CoREST-mediated silencing of viral genes by dissociating histone deacetylase 1 (HDAC1) (Gu et al., 2005). ICP0 targeting of ring finger protein 168 (RNF8) and ring finger protein 168 (RNF168), which are ubiquitin ligases mediating histone 2A (H2A) and histone 2A member X (H2AX) ubiquitination in the DDR pathway, leads to failure to recruit host DDR repair factors (Lilley et al., 2010; Chaurushiya et al., 2012). ICP0 also scrambles innate immune pathways through degradation of DNA-dependent protein kinase catalytic subunit (DNA-PKcs) (Burleigh et al., 2020). Because of these mechanisms that antagonize host repressive functions, ICP0 is important for efficient initiation of lytic infection, and replication of HSV-1 is severely impaired without ICP0 or its RING finger domain in multiple cell types especially at low multiplicities of infection (MOIs) (Cai and Schaffer, 1992; Everett et al., 2004).

Although research about ICP0 targets was mostly carried out in non-neuronal cells, there is much evidence that ICP0 is important for HSV-1 neuronal infection. For example, ICP0 mutant viruses were severely impaired in establishment of latency and reactivation in ganglionic neurons or tissues (Wilcox et al., 1997; Halford and Schaffer, 2001; Thompson and Sawtell, 2006) and delivery of ICP0 by adenovirus to ganglionic neurons stimulated HSV-1 reactivation from latency (Halford et al., 2001). An HSV-1 mutant with increased ICP0 expression due to mutations in binding sites of a neuron-specific miRNA showed increased overall lytic gene expression in acutely and latently infected mouse trigeminal ganglia (TG) (Pan et al., 2014; Sun et al., 2021). ICP0 deficiency also affected the levels of heterochromatin on viral genes in latently infected ganglia (Raja et al., 2016).

High-throughput proteomic approaches have been successfully employed to discover host interacting proteins and substrates of ICP0. One study characterized changes in cellular SUMO2 proteome upon HSV-1 infection in hepatocytes (Sloan et al., 2015). A recently published study compared wild-type (WT) virus and a mutant lacking functional ICP0 in the proteome associated with viral DNA (Kim et al., 2021). We also notice three studies of the ICP0 interactome, one in ICP0-transfected 293T cells (Sato et al., 2016b), one in HSV-1–infected Hep-2 cells (Sato et al., 2016a), and one in HSV-1–infected 293T cells (Conwell et al., 2015). However, these studies used only a few non-neuronal cell lines. Given that the functions of ICP0 are cell type–dependent (Rodriguez et al., 2020), ICP0 targets in different cell types, especially in neuronal cells, need to be further investigated. Therefore, we undertook proteomic approaches to investigate ICP0 targets with emphasis on neuronal cells. Besides the commonly performed interactome analysis, comprehensive protein ubiquitination investigation for this ubiquitin E3 ligase should greatly facilitate target identification. Because ubiquitin forms an isopeptide bond with its substrate and generates a signature di-glycine modification on lysine following trypsin digestion, immunoprecipitation (IP) using di-glycine antibody has been widely applied to enrich peptides with ubiquitin remnants before mass spectrometry (MS)–based shotgun proteomics survey of ubiquitinated proteins (hereafter referred to as ubiquitinome) (Udeshi et al., 2013; Fulzele and Bennett, 2018). Recently, di-glycine enrichment has been coupled with tandem mass tag (TMT)–based multiplexed isotopic labeling for parallel quantitative ubiquitinome analysis (Rose et al., 2016; Udeshi et al., 2020). In this work, we employed this method in combination with immunoprecipitation-based interactome analysis to identify ICP0 targets in neuronal cells followed by assessing the effects of these targets on viral replication and the host interferon (IFN) response.

## Results

### ICP0 Was Confirmed to Be Important for Herpes Simplex Virus 1 Replication in Primary Neurons and Neuro-2a Cells

**FIGURE 1:**
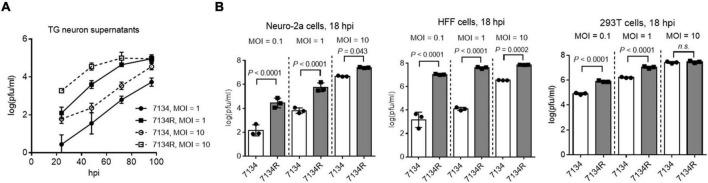
ICP0 is important for HSV-1 replication in neuronal cells. **(A)** Primary mouse TG neurons were infected with 7134 or 7134R at an MOI of 1 or 10 as indicated. At the indicated times post-infection, supernatants were collected for virus titration. **(B)** Neuro-2a (left), HFF (middle), and 293T (right) cells were infected with 7134 or 7134R at the indicated MOI and harvested at 18 hpi for virus titration. Data were analyzed by one-way ANOVA with Sidak’s multiple comparisons tests and are presented as mean values ± standard deviations (SD).

To better understand the importance of ICP0 in HSV-1 neuronal replication, we first examined replication of an ICP0-null virus, 7134, relative to its rescued derivative, 7134R in cultured primary neurons isolated from mouse TG. The isolation was performed with density gradient centrifugation following dissociation of the tissues to obtain relatively pure neurons (Katzenell et al., 2017). Viral titers were determined by plaque assays in human bone osteosarcoma (U2OS) cells, which are permissive for ICP0-null virus replication. After infection of the primary neurons, the supernatant titers of 7134 virus were ∼2 logs lower than those of 7134R virus at both MOIs of 1 and 10 from 24 to 72 h post-infection (hpi) (Figure 1A). For proteomic analysis, which requires a large number of cells, we considered using Neuro-2a cells derived from mouse brain neuroblastoma cells because results from these cells could often be recapitulated in mouse models (Pan et al., 2014; Sun et al., 2021). After infection of Neuro-2a cells, 7134 virus replicated to ∼2 logs lower titers than 7134R at MOIs of 0.1 and 1 at 18 hpi (Figure 1B). However, the defect of 7134 virus was modest at the high MOI of 10 in agreement with the MOI dependence of ICP0 effects commonly observed in cell lines (Cai and Schaffer, 1992; Everett et al., 2004). Under the same conditions, we also compared 7134 and 7134R in HFF (human foreskin fibroblast) and 293T (human embryonic kidney) cells. The defects of 7134 virus were more severe in HFF cells but moderate in 293T cells consistent with the known cell type–specific role of ICP0. These results substantiated the importance of ICP0 and suggested that Neuro-2a cells could be used to investigate the important interactions with ICP0 in neuronal cells.

### Construction and Characterization of Flag-ICP0 Virus

**FIGURE 2:**
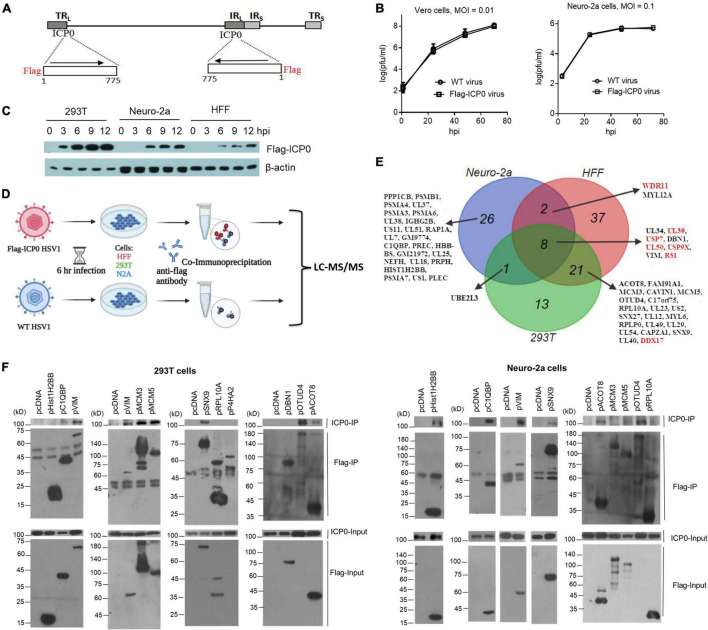
ICP0-interactome analysis. **(A)** Diagram of recombinant Flag-ICP0 virus. The horizontal line at the top represents HSV-1 genome and the boxes labeled with TR_L_, IR_L_, TR_S_, and IR_S_ represent the repeated sequences. At the bottom are expanded ICP0 gene regions with the inserted N-terminal Flag tags indicated. **(B)** Virus growth curves of WT and Flag-ICP0 virus in Vero (left) and Neuro-2a (right) cells after infection at an MOI of 0.01 and 0.1, respectively. Data are presented as mean values ± SD. **(C)** Neuro-2a, HFF, and 293T cells were infected with Flag-ICP0 virus at an MOI of 10 and cells were lysed at the indicated time points for examination of Flag-ICP0 expression by Western blots with anti-Flag and anti-β-actin antibodies. **(D)** Schematic diagram of the procedures for ICP0 interactome analysis. Neuro-2a, 293T, and HFF cells were infected with WT (control) or Flag-ICP0 virus for 6 h at an MOI of 20 with three biological replicates. Proteins were immunoprecipitated by the anti-Flag antibody and examined by liquid chromatography with tandem mass spectrometry (LC-MS/MS). **(E)** Venn diagram showing candidate ICP0-interacting proteins identified by the interactome analysis in the three cell types. Previously reported ICP0-interacting proteins are marked in red. **(F)** 293T (left) or Neuro-2a (right) cells in each 100-mm plate were co-transfected with 7.5 μg of an empty vector (pcDNA) or a plasmid expressing the indicated protein linked to a Flag-tag and 7.5 μg of a plasmid expressing untagged ICP0 for 48 h. After the cells were lysed, the indicated proteins were immunoprecipitated by an anti-Flag antibody and analyzed by Western blots for ICP0 and the corresponding proteins using an ICP0 and Flag antibody, respectively. Results from immunoprecipitated samples (IP) were shown in the upper panels and those from the lysates (Input) were shown in the lower panels. Note that prolyl 4-hydroxylase subunit alpha 2 (P4HA2) and OTUD4 expression from the plasmids were not detected in the input samples due to low expression but were clearly detected in the IP samples.

To facilitate interactome analysis in infected cells, we constructed Flag-ICP0 virus that expresses N-terminal Flag-tagged ICP0 using bacterial artificial chromosome (BAC) technology on the basis of WT BAC virus described previously (here referred to as WT virus) (Figure 2A; Pan and Coen, 2012). The Flag-tag expressing sequence was inserted into both copies of the ICP0 gene. The resulting Flag-ICP0 virus replicated with WT kinetics in Vero and Neuro-2a cells (Figure 2B). A Flag antibody readily detected ICP0 expression from the virus in 293T, HFF, and Neuro-2a cells (Figure 2C). ICP0 expression reached plateaus in all the three cell lines at around 6 hpi, so we decided to use 6 h as the time point to collect samples for the following interactomic experiments.

### ICP0 Interactome Analysis in Herpes Simplex Virus 1–Infected Cells

To analyze the ICP0 interactome, we infected Neuro-2a, HFF, and 293T cells with Flag-ICP0 or WT virus for 6 h before co-immunoprecipitation (Co-IP) with a Flag antibody followed by protein identification by liquid chromatography with tandem mass spectrometry (LC–MS/MS) (Figure 2D). After applying selection criteria (see section “Materials and Methods”), we identified 37, 68, and 43 potential ICP0-interacting proteins in Neuro-2a, HFF, and 293T cells, respectively, amounting to a total of 108 candidate proteins (Figure 2E and Supplementary Table S1). Eight proteins were found in all three cell lines: viral proteins UL50, UL39, RS1, and UL34 and host proteins USP7, USP9X, vimentin (VIM), and DBN1. The interactome overlaps very well between 293T and HFF cells in that 67% of proteins identified in 293T cells were also identified in HFF cells and 43% of proteins identified in HFFs were also identified in 293T cells. However, the overlap between mouse Neuro-2a cells and any of the human cell lines was poor. This might imply that the differences in ICP0 interactome between host species might be greater than those between cell types. Therefore, we consider the proteins identified in all three cell lines as the ICP0 interactome group for subsequent analyses. Functional enrichment analysis of this group revealed that ICP0 interacted with a host protein network related to several biological function clusters including deubiquitination, proteasome, cytoskeleton, translation elongation, mRNA processing, and protein transport (Supplementary Figure S1). The group also included many previously reported ICP0 targets such as viral proteins UL50 (Conwell et al., 2015), UL39 (Conwell et al., 2015), and RS1 (ICP4) (Yao and Schaffer, 1994; Kalamvoki et al., 2008) and host proteins USP7 (Everett et al., 1997), USP9X (Sato et al., 2016a), PRKDC (Parkinson et al., 1999), DDX17 (Mallon et al., 2012), and WDR11 (Taylor and Mossman, 2015).

### Validation of Novel Interactions With ICP0

To validate the proteomics data, we selected some top novel host candidates according to Flag-ICP0/WT ratios. With priorities given to the candidates identified in at least two cell lines, we selected a total of 17 top candidates and cloned their human genes into expressing plasmids with Flag tags. After co-transfection of plasmids with an ICP0 expressing plasmid into Neuro-2a and 293T cells, we performed IP in a reverse way relative to the interactome analysis. We confirmed that untagged ICP0 could be pulled down by Flag-tagged complement C1q binding protein (C1QBP), VIM, sorting nexin 9 (SNX9), acyl-coenzyme A thioesterase 8 (ACOT8), and ovarian tumour domain-containing protein 4 (OTUD4) in both Neuro-2a and 293T cells (Figure 2F). We note that although SNX9, ACOT8, and OTUD4 were not identified in the interactome analysis in Neuro-2a cells possibly due to limited sensitivity of MS or the human-mouse differences, the reverse Co-IP showed that their human forms were all able to interact with ICP0 in Neuro-2a cells. Therefore, these proteins were included in the following functional studies. Hist1H2BB co-precipitated with ICP0 in Neuro-2a but not 293T cells. MCM3 and MCM5 co-precipitated with ICP0 in 293T but not Neuro-2a cells. Therefore, about half of the candidates tested (and 7% of the interactome group) were able to coimmunoprecipitate with ICP0 in at least one cell line. Other proteins that we examined, including heterogeneous nuclear ribonucleoprotein M (HNRNPM), prolyl 4-hydroxylase subunit alpha 2 (P4HA2), ribosomal protein L10a (RPL10A), myosin light chain 12A (MYL12A), protein phosphatase 1 catalytic subunit beta (PPP1CB), caveolae associated protein 1 (CAVIN1), drebrin 1 (DBN1), proteasome 20S subunit alpha 3 (PSMA3), and proteasome 20S subunit alpha 6 (PSMA6), could not reproducibly co-immunoprecipitate with ICP0 in either cell line (data not shown).

### ICP0-Interacting Protein SNX9 Restricted Replication of an ICP0-Null Virus in Neuro-2a Cells

**FIGURE 3:**
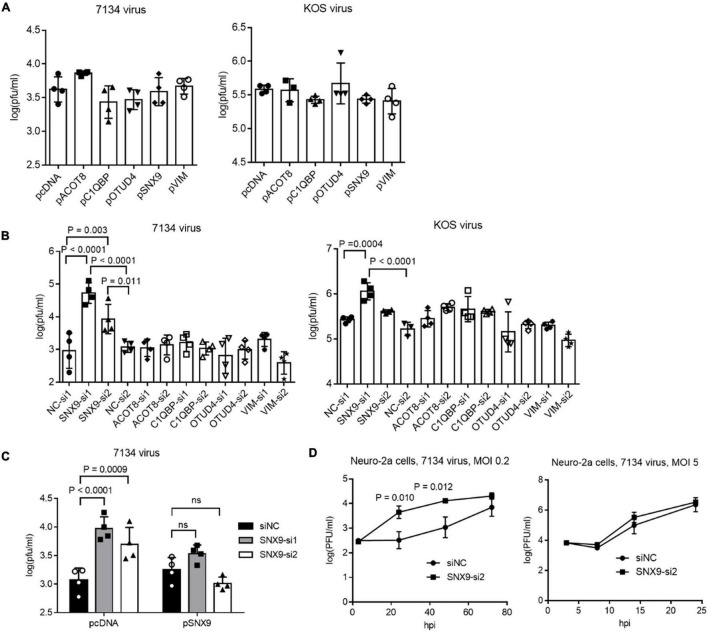
Effects of ICP0-interacting proteins on HSV-1 replication in Neuro-2a cells. **(A)** Neuro-2a cells were transfected with the indicated plasmids (400 ng/ml) for 40 h and then infected with 7134 (left) or KOS (right) virus for 48 h at an MOI of 0.2 before the cells were harvested for virus titration by plaque assays. **(B)** Neuro-2a cells were transfected with the indicated siRNAs (80 nM) for 40 h and then infected with 7134 (left) or KOS (right) virus for 48 h at an MOI of 0.2 before the cells were harvested for virus titration. NC, negative control. **(C)** Neuro-2a cells were transfected with the indicated siRNAs (80 nM) for 17 h and then transfected with the indicated plasmids (600 ng/ml) for 33 h before infection with 7134 virus at an MOI of 0.2 for 44 h. The cells were then harvested for viral titration. **(D)** Neuro-2a cells were transfected with a negative control siRNA or an siRNA against SNX9 for 40 h before infection with 7134 virus at an MOI of 0.2 (left) or 5 (right). The cells were harvested at the indicated times for virus titration. Data were analyzed by one-way ANOVA with Dunnett’s multiple comparisons tests **(A,B)**, two-way ANOVA with Sidak’s multiple comparisons tests **(C)**, or two-tailed unpaired *t*-tests **(D)**. Data are presented as mean values ± SD.

To explore the functions of the ICP0-interacting proteins, we focused on the five proteins that co-immunoprecipitated with ICP0 in both 293T and Neuro-2a cells, including C1QBP, VIM, SNX9, ACOT8, and OTUD4. Besides using the plasmids to overexpress the proteins, we designed at least two siRNAs to knock down each protein. After evaluating the knockdown efficiencies, two siRNAs with the highest efficiencies for each protein was selected along with two negative control siRNAs. Efficient knockdown by the selected siRNAs and overexpression by the plasmids in Neuro-2a cells were confirmed in Western blots (Supplementary Figure S2). We then infected the cells after transfection of the plasmids or siRNAs. Overexpression of none of these proteins had a significant effect on replication of 7134 or KOS virus (Figure 3A). However, both siRNAs against SNX9 resulted in significantly increased 7134 virus titers relative to both control siRNAs in Neuro-2a cells, although only one of them (SNX9-si1) showed modestly increased KOS titers (Figure 3B). We then performed co-transfection of the siRNAs together with plasmids. Although both siRNAs against SNX9 significantly increased 7134 virus titers in the presence of the control plasmid, they could not do so in the presence of the SNX9 expressing plasmid (Figure 3C), suggesting that the increased replication was caused by specific knockdown of SNX9. Growth curve analysis showed that even the less effective SNX9 siRNA (SNX9-si2) significantly increased replication kinetics of 7134 virus at an MOI of 0.2 but it had no effect at an MOI of 5 (Figure 3D). Therefore, endogenous SNX9 restricted HSV-1 replication at a low MOI.

### ICP0 Ubiquitinome Analysis in Infected Neuro-2a Cells

**FIGURE 4:**
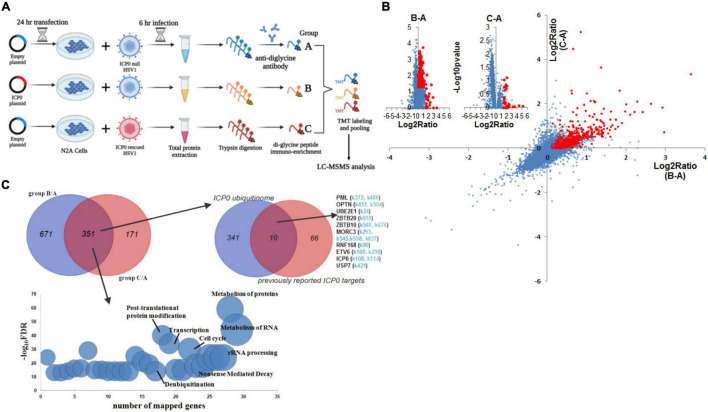
ICP0 ubiquitinome analysis in Neuro-2a cells. **(A)** Schematic diagram of the experimental design for ICP0 ubiquitinome analysis. **(B)** Potential ICP0 target sites (red) showing higher ubiquitination levels in group B than A (*x*-axis) or those showing higher ubiquitination levels in group C than A (*y*-axis) according to quantitative mass spectrometry. Individual volcano diagrams showing B-A and C-A comparisons were also displayed in the upper left corner. **(C)** Potential ICP0 ubiquitination target proteins identified by comparing groups B and A as well as comparing groups C and A. The overlap of the two comparisons represents 351 candidate targets for subsequent analysis. The pathways enriched in these proteins are shown in the bottom graph. The upper right Venn diagram shows the overlap of our ICP0 ubiquitinome results and previously reported ICP0 substrates or interacting proteins. For the targets in our data that were also previously reported, potential ubiquitination sites on lysine differentially represented between ICP0-positive and negative cells in our data are indicated in light blue.

To identify ubiquitination substrates of ICP0, we compared the ubiquitinome during HSV-1 infection in the presence versus absence of ICP0. Absence of ICP0 was achieved using the ICP0-null virus 7134, which was compared with 7134R. To increase the stringency of the experiment, we also considered rescuing the absence of ICP0 by transfection because real targets should show differences in comparisons both between 7134 and 7134R viruses and between ICP0 transfected and untransfected cells. Therefore, we designed the following three groups: group A, control transfection + 7134 virus infection; group B, ICP0 transfection + 7134 virus infection; group C, control transfection + 7134R virus infection. Only ubiquitination events that were induced both by transfected ICP0 (comparing B and A) and by ICP0 expressed from the virus (comparing C and A) were considered, which should greatly reduce frequencies of false-positive discoveries. Neuro-2a cells were transfected for 24 h and then infected for 6 h at an MOI of 20 before total protein was digested by trypsin and immunoprecipitated with ubiquitin remnant K-ε-GG antibody followed by TMT labeling and LC-MS/MS analysis (Figure 4A). Overall, ratios of protein quantities in group B versus A (B/A) and group C versus A (C/A) comparisons correlated well with each other (Figure 4B). After applying selection criteria for the differences and mapping these sites to proteins, we identified 1,022 proteins in the B/A comparison and 522 proteins in the C/A comparison. Importantly, a large fraction of these proteins (351 proteins) was commonly identified by both comparisons. These proteins were considered as potential ICP0 substrates (Figure 4C and Supplementary Table S1). Functional enrichment analysis showed that the potential host substrates are enriched in multiple pathways, including metabolism of RNA/proteins, rRNA processing, cell cycle, and deubiquitination (Figure 4C). Nine proteins were previously reported to be potential host substrates of ICP0 including USP7 (Boutell et al., 2005), PML (Cuchet-Lourenco et al., 2012), OPTN (Waisner and Kalamvoki, 2019), SUMO2 (Sloan et al., 2015; Drayman et al., 2017), UBE2E1 (Vanni et al., 2012), ZBTB10 (Sloan et al., 2015), MORC3 (Sloan et al., 2016), RNF168 (Lilley et al., 2010), and ETV6 (Sloan et al., 2015). Their potential ubiquitination sites determined by our MS data are listed in Figure 4C. In addition, the candidate substrate proteins were clustered in several superfamilies classified by conserved domains such as nucleoside triphosphate hydrolase, RNA helicase, zinc finger, RNA binding, and ubiquitin (Supplementary Table S2). Regarding viral proteins, out of 55 viral proteins with detected ubiquitination, seven proteins, namely, UL12, UL21, UL5, UL51, UL54, UL9, and US3, showed increased ubiquitination in the presence of ICP0 (Supplementary Figure S3), indicating that they might be potential viral substrates of ICP0.

### OTUD4 and VIM Could Be Ubiquitinated by ICP0

**FIGURE 5:**
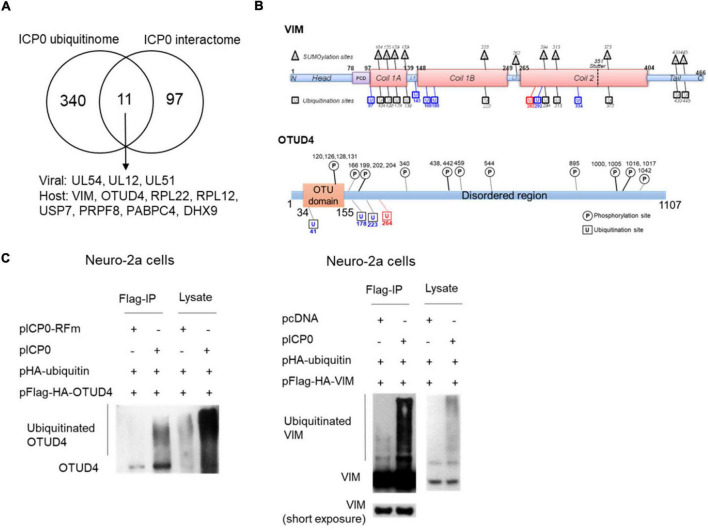
ICP0-mediated ubiquitination of OTUD4 and VIM. **(A)** Venn diagram showing 12 proteins identified by both ICP0 interactome and ubiquitinome analyses. **(B)** Summary of ubiquitination sites on VIM (upper) and OTUD4 (lower). Schematic representation of functional domains of VIM and OTUD4 decorated with previously documented SUMOylation (for VIM, in triangles) or phosphorylation (for OTUD4, in circles) sites and newly identified ubiquitination sites (in squares). L, linker region; PCD, pre-coiled domain. Blue indicates sites with ubiquitination but not SUMOylation. Red indicates the ubiquitination site potentially targeted by ICP0. **(C)** Neuro-2a cells in each 100-mm plate were co-transfected with 5 μg of a plasmid expressing Flag-HA-tagged OTUD4 (left) or VIM (right), 2 μg of a plasmid expressing HA-tagged ubiquitin, and 3 μg of a plasmid expressing ICP0 or its RING finger mutant (RFm) or an empty vector (pcDNA) for 48 h. Cell lysates were immunoprecipitated by an anti-Flag antibody. The lysates and immunoprecipitated samples (IP) were analyzed by Western blots for ubiquitination levels by an anti-HA antibody.

Comparison of the interactome and ubiquitinome data identified three viral proteins and eight host proteins, which interacted with ICP0 and exhibited elevated ubiquitination in the presence of ICP0, indicating that they were highly likely to be substrates of ICP0 (Figure 5A). All of these are novel potential substrates of ICP0 except for the deubiquitinase USP7, whose ubiquitination by ICP0 has been reported elsewhere (Canning et al., 2004; Boutell et al., 2005). Because host proteins VIM and OTUD4 were confirmed to robustly interact with ICP0 (Figure 2F), we focused on these two proteins for ubiquitination analysis. VIM was previously documented to be decorated with 11 SUMO2 modifications by another large-scale MS study (Hendriks et al., 2017). Our MS data detected 17 potential ubiquitination sites in VIM (Figure 5B). We note that the trypsin digestion resulted in different reminants in ubiquitinated or SUMOylated lysine, so the current LC-MSMS analysis was specific to ubiquitin modification. Interestingly, the majority of the newly identified ubiquitination sites overlapped with the SUMOylation sites, indicating possible cross-talk between ubiquitination and SUMOylation in VIM. Almost all ubiquitination and SUMOylation sites are located in the functional coil regions of VIM. Notably, the only site (K282) showing increased ubiquitination due to ICP0 presence has not been reported to be SUMOylated. The MS data also detected four potential ubiquitination sites in OTUD4, all of which are near the OTU domain and not far from the previously annotated phosphorylation sites. One of the sites (K264) showed increased ubiquitination due to ICP0 presence and, therefore, could be an ICP0 target site. To examine whether VIM and OTUD4 were ubiquitinated by ICP0, we co-transfected ubiquitin, VIM or OTUD4, and ICP0 or its control (empty vector or ring finger mutant) into Neuro-2a cells before immunoprecipitation of VIM and OTUD4 and analysis of ubiquitination levels by Western blots. Both VIM and OTUD4 exhibited substantial enhancement of ubiqutination in the presence of ICP0 relative to the respective controls, suggesting that they could be ubiquitinated by ICP0 (Figure 5C). However, transfected ICP0 did not cause decreases in VIM and OTUD4 levels indicating that ubiquitination of these proteins may not lead to degradation.

### OTUD4 Was Upregulated to Enhance Type I Interferon Expression During ICP0-Null Virus Infection

**FIGURE 6:**
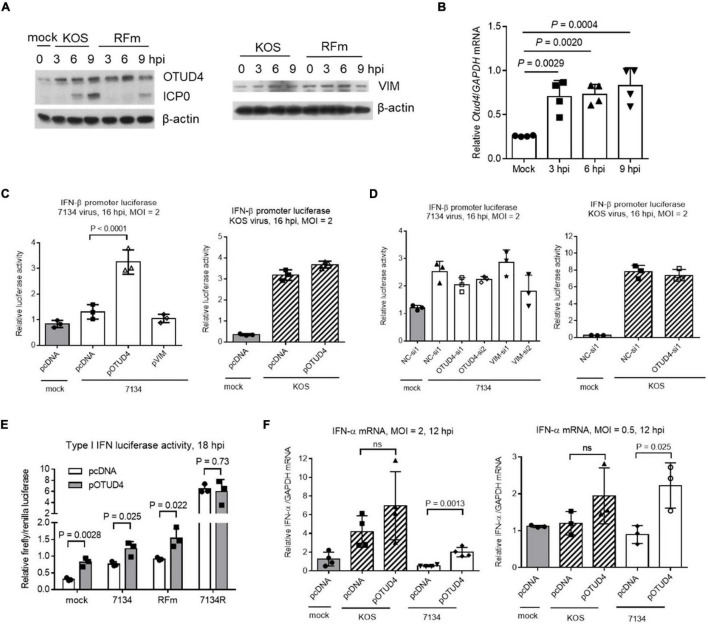
Functions of OTUD4 and VIM in Neuro-2a cells. **(A)** Cells were infected with KOS or ICP0 RING finger mutant virus (RFm) at an MOI of 20 for the indicated times before the cells were harvested for Western blot analysis for the indicated proteins. **(B)** After infection of cells with KOS at an MOI of 20, the cells were harvested at the indicated times for qRT-PCR analysis of Otud4 mRNA levels normalized to glyceraldehyde-3 phosphate dehydrogenase (GAPDH) mRNA levels. **(C)** Cells were co-transfected with the indicated plasmid (400 ng/ml), pIFN-β (80 ng/ml) (an IFN-β promoter luciferase plasmid), and pRL-TK (40 ng/ml; a control plasmid expressing renilla luciferase) for 48 h and then infected with 7134 (left) or KOS (right) at an MOI of 2 for 16 h before measurement of luciferase activities. **(D)** Neuro-2a cells were co-transfected with the indicated siRNA (80 nM), pIFN-β (80 ng/ml), and pRL-TK (40 ng/ml) for 48 h and then infected with 7134 (left) or KOS (right) at an MOI of 2 for 16 h before measurement of luciferase activities. **(E)** The assay was performed as in **(C)** except that different viruses were used. **(F)** Cells were transfected with the indicated plasmid (400 ng/ml) and then infected with the indicated virus at an MOI of 2 (left) or 0.5 (right) for 12 h before qRT-PCR quantification of *IFN-*α mRNA normalized to *GAPDH* mRNA. Data were analyzed by one-way ANOVA with Dunnett’s multiple comparisons tests **(B–D,F)** or two-tailed unpaired *t*-tests **(E)**. Data are presented as mean values ± SD.

To understand the roles of VIM and OTUD4 during HSV-1 infection, we first assessed their levels in infected Neuro-2a cells. Interestingly, we observed substantial upregulation of OTUD4 but not VIM starting from as early as 3 hpi (Figure 6A). This upregulation was independent of the E3 ligase activity of ICP0 because it was also observed during infection with an ICP0 ring finger mutant (RFm) virus. The increases in OTUD4 protein levels correlated with similar increases in its mRNA levels indicating stimulation of *Otud4* gene transcription during infection (Figure 6B). Comparison of WT and RFm viruses showed no indication of ICP0-induced degradation of OTUD4 or VIM at later times (Figure 6A). Thus, OTUD4 was upregulated during HSV-1 infection without being degraded by ICP0.

To learn more about the roles of OTUD4 and VIM, although they showed no effect on HSV-1 replication (Figures 3A,B), we wondered whether they could regulate innate immune response to infection. To test this, we first transfected Neuro-2a cells with an IFN-β promoter luciferase reporter construct together with a plasmid expressing OTUD4 or VIM before infection at an MOI of 2 for 16 h. 7134 virus only modestly stimulated luciferase expression, whereas KOS virus showed much stronger stimulation. In 7134 infected cells, overexpression of OTUD4 significantly increased luciferase activity, whereas knocking down OTUD4 or overexpressing or knocking down VIM had no significant effect (Figures 6C,D). However, in KOS-infected cells, neither overexpressing nor knocking down OTUD4 had a significant effect indicating an ICP0-dependent block of OTUD4 activity. Consistent with this, overexpressed OTUD4 increased luciferase activity during infection with an ICP0 RFm virus but had no effect during infection with 7134R virus (Figure 6E). Unexpectedly, OTUD4 also increased luciferase activity during mock infection suggesting that OTUD4 could raise the baseline expression of IFN-β in unstimulated cells. In concordance with the luciferase results, quantification of endogenous IFN-α mRNA showed that induction of IFN-α expression by KOS but not 7134 virus was obvious at an MOI of 2, and overexpressed OTUD4 significantly increased IFN-α mRNA levels in 7134 but not KOS infected cells (Figure 6F). At a lower MOI of 0.5, where induction of IFN-α expression was no longer detectable even in KOS infected cells, overexpressed OTUD4 significantly increased IFN-α mRNA levels in 7134 but not KOS virus infected cells. Therefore, OTUD4 could enhance type I IFN expression during HSV-1 infection, and this function appeared to be blocked by ICP0-dependent processes.

## Discussion

After confirming that ICP0 was important for HSV-1 lytic infection in neuronal cells, we combined quantitative interactome and ubiquitinome approaches to identify targets of ICP0 followed by validation of the interaction and ubiquitination events in Neuro-2a cells. Functional studies on the validated targets established SNX9 as a restriction factor and OTUD4 as a regulator of type I IFN production and provided evidence that the functions of these proteins might be modulated in an ICP0-dependent manner.

The use of two proteomic approaches allowed us to dissect binding and ubiquitination events, which may not always be coupled. Binding interactions may function in ways independent of ubiquitination, and some ubiquitin modifications might result from transient interactions that were not detected after immunoprecipitation. Besides, there were likely to be false positive candidates from each approach. These might be the reason for the low number of proteins commonly identified by the two approaches. However, the candidates identified by both approaches were likely to be valid substrates. Indeed, we confirmed that both OTUD4 and VIM could be ubiquitinated by ICP0.

Our data recapitulated many of the previous reported ICP0-interacting proteins or substrates. Two important known substrates are USP7 and PML. Whereas USP7 was identified in both interactome and ubiquitinome analyses, PML was identified only by the ubiquitinome analysis indicating that the interaction between ICP0 and PML might be too transient to be caught by immunoprecipitation. USP7 is a deubiquitinase that can inhibit ICP0 autoubiquitination, thereby leading to ICP0 stabilization (Boutell et al., 2005, 2011). The role of PML in restriction of viral gene transcription and its antagonism by ICP0 have been well documented (Everett et al., 1998; Zheng et al., 2016; Alandijany et al., 2018). Our data suggest that these processes likely occur in neuronal cells too.

Functional studies on selective novel interaction partners and/or substrates of ICP0 revealed two potential host restrictive factors SNX9 and OTUD4. We note that SNX9 and OTUD4 were identified in the interactome analysis in 293T and HFF but not Neuro-2a cells. This could be due to low endogenous levels of OTUD4 and SNX9 in Neuro-2a cells or the possibility that their mouse forms simply do not interact with ICP0. Nevertheless, we have solid evidence that the human forms of these proteins interact with ICP0 because their endogenous proteins were identified in the ICP0 interactome in both 293T and HFF cells and their transfection resulted in detectable interaction with ICP0 in both 293T and Neuro-2a cells. It will be important to test in future whether they interact with ICP0 in human neurons. SNX9 restricted HSV-1 replication and OTUD4 could induce type I IFN production, which might potentially restrict HSV-1 replication. Our results that these proteins had effects during infection with the ICP0-null but not WT virus indicated that their activities might be modulated by ICP0 or ICP0-induced processes. These regulatory pathways are likely additive to the known pathways targeted by ICP0 to promote lytic infection, like the PML-mediated pathway. SNX9 is a member of the sorting nexin family involved in intracellular trafficking and clathrin-mediated endocytosis (Bendris and Schmid, 2017), yet its role in viral infection has not been reported. Another member of the SNX family, SNX27 has been reported to suppress SARS-CoV-2 infection by inhibiting viral lysosome/late endosome entry (Yang et al., 2022). Therefore, we speculate that SNX9 might regulate HSV-1 entry through the endocytic pathway. Like USP7, OTUD4 is a deubiquitinase that cleaves ubiquitin chains from their targets and thereby counteracts ubiquitination mediated by E3 ligases. OTUD4 has been reported to preferentially cleave lysine48–linked poly ubiquitin chains (Mevissen et al., 2013). Its function is implicated in the repair of DNA alkylation damage (Zhao et al., 2015). Interestingly, one study reported that OTUD4 is induced during RNA virus infection to promote antiviral response (Liuyu et al., 2019), which is analogous to the role of OTUD4 in HSV-1 infection that we report here. That study showed that OTUD4 deubiquitinates and stabilizes MAVS, thereby activating downstream signaling in the RIG-I like receptor RNA sensing pathway. Because this pathway is known to function during HSV-1 infection too (Liu et al., 2021), it is possible that OTUD4 functions by a similar mechanism during HSV-1 infection although this hypothesis needs to be tested in future. VIM is an intermediate filament protein that participates in many biological processes including cytoskeletal assembly and immune response (Ramos et al., 2020). We found no evidence of its regulation of HSV-1 replication or IFN expression, and we found no report of its regulation of the IFN response. However, VIM has been reported to interact with NLRP3 and activate NLRP3 inflammasome in macrophages (dos Santos et al., 2015). Consistent with its role in inflammation, VIM was reported to be upregulated in cerebralspinal fluid from children with enterovirus-associated meningoencephalitis (Sun et al., 2020). Such functions in inflammation might not manifest in changes in virus production in cell culture and might require investigation in animal models.

Because many of our experiments were conducted in Neuro-2a cells, this study helps understand host interactions with ICP0 in the neuronal environment. However, we caution that Neuro-2a is a mouse cell line. The interactions identified here may not represent interactions occurring in human non-dividing neurons which HSV-1 infects in nature. Future studies are needed to examine these interactions and the associated regulatory events in human neurons.

## Materials and Methods

### Cells and Viruses

Vero, 293T, Neuro-2a, and HFF cells were obtained from American Type Culture Collection and cultured as previously reported (Pan et al., 2019). The method for culturing primary mouse TG neurons was described previously (Katzenell et al., 2017; Sun et al., 2021). HSV-1 strains KOS and its derivatives including 7134, 7134R (Cai and Schaffer, 1989), and WT-BAC (Pan and Coen, 2012) were described previously. HSV-1 Flag-ICP0 virus was prepared according to a previously described protocol using BAC technology (Tischer et al., 2006; Pan et al., 2014). Briefly, PCR-amplified zeocin-resistance (*Zeo*) cassette containing the DYKDDDDK (Flag)-coding sequence was inserted into one of the two copies of *ICP0* gene just downstream of the start codon. Then, the PCR-amplified kanamycin-resistance (*Kan*) cassette containing the Flag-coding sequence and a homologous sequence was inserted into the other copy of *ICP0*. The *Kan* cassette was then removed by recombination *via* the homologous sequence, resulting in markerless insertion of the Flag-coding sequence into the second *ICP0* gene copy. At the location of the first *ICP0* gene copy, the *Zeo* cassette was then replaced with the *Kan* cassette, which was subsequently removed as for the other *ICP0* copy. In each step, correct colonies were selected by PCR and sequencing as well as restriction fragment length polymorphism following digestion with *Eco*RI and *Hin*dIII. BAC DNA was purified and transfected into Vero cells for Flag-ICP0 virus production. Primers used are listed in Supplementary Table S3. HSV-1 infection of cells and plaque assays for viral titer measurements were performed as previously described (Pan et al., 2019).

### Plasmids

FLAG-HA-pcDNA3.1 (Addgene, #52535) was used to construct plasmids expressing hemoglobin, beta adult s chain (HBB-BS), VIM, unique long 50 (UL50), ubiquitin conjugating enzyme E2 L3 (UBE2L3), C1QBP, histone H2B type 1-B (Hist1H2BB), DEAD box RNA helicase 17 (DDX17), HNRNPM, DBN1, PSMA6, P4HA2, minichromosome maintenance complex component 3 (MCM3), minichromosome maintenance complex component 5 (MCM5), RAS-related protein 1a (RAP1A), OTUD4, SNX9, ACOT8, RPL10A, MYL12A, PPP1CB, CAVIN1, and PSMA3 with Flag-HA tags. HA-ubiquitin was constructed on the basis of HA-pcDNA3.1, which was modified from FLAG-HA-pcDNA3.1. The firefly luciferase plasmid with IFN-β promoter (pIFN-β) and Renilla luciferase plasmid pRL-TK were kind gifts of Professor Chunfu Zheng at Fujian Medical University. To construct pICP0, the complete coding sequence of ICP0 was PCR-amplified from ICP0-WT plasmid (pRS-1) (Sandri-Goldin et al., 1987) and inserted into pcDNA3.1 without Flag or HA tags. Primers used for PCR were listed in Supplementary Table S3.

### Transfection

All transfections were conducted using Lipofectamine 3000 (Thermo Fisher, Waltham, MA, United States) according to the manufacturer’s instructions. The siRNAs were synthesized by RiboBio. Their target sequences are as follows: ACO8-si1, GACCCTAACCTTCACAAGA; ACOT8 -si2, CCAAACAGATGTTCTGGGT; C1QBP-si1, CCACCTAAT GGATTTCCTT; C1QBP-si2, CATTTGATGGTGAGGAGGA; OTUD4-si1, GCTTCTTCATGCTGAATAT; OTUD4-si2, CCAG CAGAACATATACCTT; SNX9-si1, GTGGGTTTATCCTACCT CT; SNX9-si2, CGGATCTATGATTACAACA; VIM-si1, CAGA CAGGATGTTGACAAT; VIM-si2, CTTCTCAGCATCACGA TGA.

### Western Blot Analysis

Cells were scraped into sodium dodecyl sulfate (SDS) loading buffer (40% glycerol, 0.24 M Tris-HCl, pH 6.8, 8% SDS, 0.04% bromophenol blue, 5% β-mercaptoethanol) and heated at 95°C for 5 min before being loaded onto SDS polyacrylamide gels for Western blot analysis. The following antibodies and dilutions were used: Flag antibody (MBL, Tokyo, Japan, M185-3L), 1:5,000; ICP0 antibody (Santa Cruz, 11060), 1:2,000; β-tubulin antibody (Sungene Biotech, Tianjin, China, KM9003), 1:5,000; rabbit HA antibody (Sangon, Shanghai, China, D110004), 1:1,000; β-actin antibody (Abclonal, Wuhan, China, Ac026), 1:5,000; ICP4 antibody (Abcam, Cambridge, England, ab6514), 1:5,000; gC antibody (Fitzgerald, North Acton, MA, United States, 10-H25A), 1:1,000; ICP27 antibody (Virusys, Taneytown, MD, United States, 1113), 1:5,000; ACOT8 rabbit polyclonal antibody (Sangon, D221492), 1:500; C1QBP rabbit polyclonal antibody (Sangon, D152901), 1:1,000; OTUD4 rabbit polyclonal antibody (Abclonal, A15229): 1:1,000; SNX9 rabbit polyclonal antibody (Sangon, D154017), 1:1,000; VIM rabbit polyclonal antibody (Sangon, D220268), 1:3,000; horseradish peroxidase (HRP)-conjugated goat anti-mouse (SouthernBiotech, Birmingham, AL, United States, 1030-05), 1:2,000; goat anti-rabbit antibodies (SouthernBiotech, 4030-05), 1:2,000.

### Sample Preparation for ICP0 Interactome Profiling

Neuro-2a cells (2 × 10^7^) in a T150 flask were infected with Flag-ICP0 or WT virus at an MOI of 20 and harvested at 6 hpi. Cells were first washed twice in ice-cooled phosphate buffered saline (PBS) and then lysed in 1 ml of lysis buffer [50 mM HEPES-KOH (pH 7.4), 1% Triton X-100, 150 mM NaCl, 10% glycerol, and 2 mM EDTA plus one Complete EDTA-free protease inhibitor tablet (Roche) per 50 ml] for 1 h. Supernatants were first incubated with 25 μl of mouse immunoglobulin G (IgG) for 1 h at 4°C to remove non-specific binding proteins and then incubated with 50 μl of Pierce Anti-DYKDDDDK Magnetic Agarose (ThermoFisher, A36797) for 2 h. The agarose was washed four times with lysis buffer for a total of 1 h and then washed four times with PBS to remove Triton X-100. Proteins were eluted in 300 μl of elution buffer (0.1 M glycine, pH 2.8) at room temperature for 7 min and neutralized with 30 μl of Neutralization Buffer: (1 M Tris, pH 8.5). Experiments in HFF and 293T cells were conducted in the same way except that an MOI of 10 was used for infection. Immunoprecipitated proteins were reduced with 10 mM dithiothreitol (DTT) for 45 min at 30°C and alkylated with 30 mM iodoacetamide (IAA) for 30 min at room temperature in the dark. Proteins were then cleaned up by acetone precipitation and resuspended in 50 mM ammonium bicarbonate. Proteins were digested by trypsin (Promega, Madison, WI, United States) at a ratio of 1:50 (trypsin:protein) at 37°C overnight. Tryptic peptides were acidified by trifluoroacetic acid (TFA) and were desalted using a hydrophilic-lipophilic-balanced (HLB) microplate (Waters, Milford, CT, United States). The microplate was conditioned with 200 μl of acetonitrile (ACN), followed by 200 μl of 60% ACN and then by 200 μl of 0.1% TFA twice. The sample was loaded to the microplate and then washed three times with 200 μl of 0.1% TFA. Desalted peptides were eluted with 60% ACN and lyophilized in a vacuum lyophilizer (LABCONCO) before LC-MS/MS analysis.

### Sample Preparation for Quantitative Ubiquitinome Investigation

The following three groups were compared to determine the effects of ICP0 on ubiquitinome: Group A, pcDNA3.1 transfection + ICP0 null virus infection; Group B, ICP0 plasmid transfection + ICP0 null virus infection and group; and Group C, pcDNA3.1 transfection + ICP0-rescued virus infection. Neuro-2a cells in a 100-mm dish were transfected with ICP0 or pcDNA3.1 plasmid for 24 h before infection with 7134 virus (ICP0-null) or 7134R virus (ICP0-rescued) at an MOI of 20. After the 1-h incubation for virus absorption, fresh medium was added with 10 μM MG-132 to inhibit proteasome-dependent degradation. The cells were harvested at 6 hpi in urea lysis buffer (20 mM Hepes, pH 8.0, 9 M urea). After sonication and centrifugation, the supernatants were kept.

Proteins (10 mg) were reduced with 10 mM DTT for 45 min at 30°C and subsequently alkylated with 30 mM IAA for 30 min at room temperature in the dark. Lysis buffer was then replaced by 50 mM triethylammonium bicarbonate (TEAB) using Zeba Spin Desalting Columns. Proteins were digested by L-1-tosylamido-2-phenylethyl chloromethyl ketone (TPCK)-treated trypsin (Sigma, Saint Louis, Mo, United States) at a ratio of 1:40 (trypsin:protein) at 37°C overnight. Tryptic peptides were acidified by TFA and desalted using a 500 mg C18 Sep-Pak SPE cartridge (Waters). C18 cartridges were conditioned with 4 ml of ACN, followed by 4 ml of 50% ACN + 0.1% TFA and then by 4 ml of 0.1% TFA. The sample was loaded to the C18 cartridge and then washed three times with 4 ml of 0.1% TFA. Desalted peptides were eluted with 50% ACN + 0.1% formic acid (FA) and lyophilized in a vacuum lyophilizer (LABCONCO).

### Enrichment of K-ε-GG Peptides

Di-Gly–modified peptides derived from ubiquitinated proteins were enriched by PTMScan ubiquitin remnant K-ε-GG motif kit [cell signaling technology (CST)] according to the manufacturer’s instruction. Briefly, antibody-loaded beads were washed three times with 1 ml of pre-cooled 100 mM sodium borate (pH 9.0). Antibody was then cross-linked by 20 mM dimethyl pimelimidate for 30 min. Cross-linked antibody beads (50 μg) were mixed with tryptic peptides in cold 1 × IAP buffer for 2 h at 4°C with end-over-end rotation, followed by three washes with ice-cold IAP buffer and one wash with ice-cold water. Di-Gly–modified peptides were eluted by 50 μl of 0.15% TFA twice and dried completely by vacuum centrifugation.

### Tandem Mass Tag Isobaric Labeling

Di-Gly–modified peptides enriched from each sample were dissolved in 20 μl of 100 mM TEAB and labeled with 0.8 mg 10plex TMT Label Reagent (ThermoFisher) in 50 μl of ACN. The reaction was proceeded at room temperature for 1 h and stopped by 2 μl of 5% hydroxylamine. Supernatant was desalted by homemade C18 stage tip and dried by vacuum centrifugation.

### StageTip-Based Strong Cation Exchange Fractionation of K-ε-GG Peptides

Home-packed strong cation exchange (SCX) chromatography StageTip with Empore Cation-SR Extraction material (3M) was sequentially conditioned by 100 μl of ACN, 80% ACN + 0.1% TFA, and 5% ACN + 0.1% TFA. The combined TMT-labeled K-ε-GG peptides were resuspended in 80 μl of 5% ACN + 0.1% TFA and loaded to SCX Stagetip. The flow-through was labeled as SCX fraction 1. Peptides were then sequentially eluted with 100 μl of 5% ACN + 0.1% TFA supplemented with increasing concentration of ammonium acetate (40, 100, 200, 350, and 500 mM). The corresponding eluates were designated as SCX fractions 2, 3, 4, 5, and 6, respectively. All peptide fractions were dried by vacuum centrifugation.

### LC-MS/MS Analysis

All dried peptides were resuspended in 2% ACN and 0.1% FA and separated by nanoLC-MS/MS using an UltiMate 3000 RSLCnano system (Thermo Scientific) at the flow rate of 400 nl/min. Solvent A is 2% ACN and 0.1% FA, and solvent B is 98% ACN and 0.1% FA. Gradient elution was performed at 50°C using linear gradients of 120 min: 1–4 min with 3% (v/v) of s B, 4–6 min from 3% to 5% (v/v) B, 6–70 min from 5% to 15% (v/v) B, 70–90 min from 15% to 30% (v/v) B, 90–100 min from 30% to 80% (v/v) B, 100–110 min with 80% (v/v) B, and 110–120 min with 3% (v/v) A. The eluted peptides were analyzed by Q Exactive HF-X (Thermo Scientific) acquiring MS spectra at the resolution of 120,000 full width at half maximum (FWHM) with a mass range of 300–1,500 m/z and an AGC target of 3E6. The top 20 precursors were then fragmented by higher-energy C-trap dissociation (HCD) with collision energy approximately 32% normalized collision energy (NCE) and tandem mass spectrometry (MS2) spectra were acquired at the resolution of 45,000 FWHM.

### Mass Spectrometry Data Analyses

All MS raw data were loaded into MaxQuant (version 1.6.2.10) and searched against human or Mouse UniProtKB database (November 2019) supplemented with HSV1 sequences, with the automatic reverse database on target-decoy search mode. MS2-based isobaric labeling using 10plex TMT tags were configured only for the ubiquitinome analysis. Variable modifications including oxidation (M) (+15.99491 Da) and acetyl (protein N-term) (+42.01056 Da) were specified for ubiquitinome, proteome, and ICP0-interactome analyses, whereas GlyGly(K)_10plex_TMT (+ 343.20586 Da) was specified only for the ubiquitinome analysis. Carbamidomethyl (C) (+ 57.02146 Da) was set as the fixed modification. Trypsin was set as digestion mode with two maximum missed cleavage sites. We used 20 ppm in the first search ion tolerance and 4.5 ppm in the main search ion tolerance. MaxQuant default setting was used for all other parameters. Peptide and protein identification were both filtered by false discovery rate (FDR) < 1%.

### Statistical and Functional Enrichment Analyses

For the interactome data sets from 293T and Neuro-2a cells, potential ICP0-interacting proteins were selected by either fold change ≥ 5 or fold changes ≥ 1.2 with *p* < 0.05 (*t*-test) between Flag-ICP0 and WT comparison. For HFF cells, which identified a larger number of proteins, we used a more stringent criterion of fold changes > 5 and *p* < 0.1 (*t*-test). Proteins with < 2 unique peptides were discarded. The ubiquitinome data were normalized by protein levels determined by peptides without di-glycine modification. After normalization, modified sites with > 1.25 higher di-glycine levels in both B/A and C/A comparisons or with increasing di-glycine levels with FDR < 0.05 (*t*-tests) were considered as potential ICP0 target sites. To identify host pathways modulated by HSV-1 ICP0, potential ubiquitination targets of ICP0 were then submitted for functional annotation enrichment analysis in KOBAS 3 (Xie et al., 2011)^1^ against Reactome database (Jassal et al., 2020). Fisher’s exact test and Benjamini and Hochberg’s FDR < 0.01 method were chosen for significant enriched terms. Enriched term with >1000 genes in database or <10 mapped genes were discarded from analyses. Similar or redundant terms were manually removed to keep the most significantly enriched ones. All potential ICP0-interacting proteins were used to generate interaction network based on String database^2^ (Szklarczyk et al., 2019), whereas all the potential ubiquitination targets were submitted to search conserved domains by InterPro of String database (Blum et al., 2020).

### Co-immunoprecipitation for Validating Interactions

Five hours after 5 × 10^6^ Neuro-2a cells were plated in 100-mm dishes, the cells were transfected with the ICP0 expressing plasmid together with a plasmid expressing a potential interacting protein with a Flag tag. Cells were lysed in 1 ml of lysis buffer (see above) at 30 h post-transfection (hpt). The Flag-tagged proteins were immunoprecipitated as described for the ICP0 interactome analysis except that 40 μl of anti-DYKDDDDK Magnetic Agarose was used and 80 μl of 1 × SDS loading buffer was added to eluted proteins. Western blotting was conducted to detect the proteins.

### Validation of Ubiquitination

About 9 × 10^6^ Neuro-2a cells in 100-mm dishes were co-transfected with the following plasmids: 5 μg of pOTUD4 or pVIM (Flag-HA tag), 2 μg of HA-Ubiquitin and 3 μg of ICP0 or ICP0-RFm as control. Cells were lysed in lysis buffer at 48 hpt. IP was performed as described above using the anti-Flag agarose.

### Quantitative Reverse-Transcription-PCR

To quantify mRNA levels in cells, total RNA was purified using a RNA Isolation Kit (catalog # RC112, Vazyme Biotech, Nanjing, China) according to the manufacturer’s instructions. Reverse transcription and PCR were conducted using a HiScript II Q Select RT SuperMix and ChamQ Universal SYBR qPCR Kit (catalog # R233-01 and Q711-02/03, Vazyme Biotech). Data were normalized to GAPDH mRNA levels. The following primers for mouse transcripts were used: GAPDH-F: GAAGGTCGGTGTGAACGGATT; GAPDH-R: GCCTTGACTGTGCCGTTGAA; OTUD4-F: CCTC CATCTCAGGTGTCTGAAGGTCA; OTUD4-R: GGTTAGGC CCAAAAGACTGTTGTGG; IFN-α-F: CTCATTCTGCAATG ACCTCCACC, IFN-α-R: ACTTCTGCTCTGACCACCTCCC.

### Luciferase Assays

Neuro-2a cells were co-transfected with the indicated plasmid (400 ng/ml) or the indicated siRNA (80 nM), pIFN-β (80 ng/ml), and pRL-TK (40 ng/ml) for 48 h and then infected with 7134 or KOS virus for the indicated times. The cells were then lysed, and promoter activities were measured by a dual luciferase kit according to the manufacturer’s protocol (Yeasen, Shanghai, China, 11402ES60).

## Data Availability Statement

The data presented in the study are deposited in the Integrated Proteome Resources repository (https://www.iprox.cn), accession number IPX0002267000.

## Author Contributions

DP conceived the study. FH performed virus engineering, cloning, and sample preparation for mass spectrometry. FH and YD performed immunoprecipitation and ubiquitination assays. FH, DP, SC, and XY did functional analyses in cell culture. ZS, FJ, MZ, and KR performed mass spectrometry analysis. FH, ZS, and DP prepared the manuscript. All authors approved the manuscript.

## Conflict of Interest

The authors declare that the research was conducted in the absence of any commercial or financial relationships that could be construed as a potential conflict of interest.

## Publisher’s Note

All claims expressed in this article are solely those of the authors and do not necessarily represent those of their affiliated organizations, or those of the publisher, the editors and the reviewers. Any product that may be evaluated in this article, or claim that may be made by its manufacturer, is not guaranteed or endorsed by the publisher.

## Footnotes

1
http://kobas.cbi.pku.edu.cn

2
https://string-db.org

## Supplementary Material

Supplementary Figure S1 ICP0-interacting network. The functional clusters were generated by the K-mean approach based on the candidate ICP0-interacting proteins identified by the interactome analysis in all three cell lines.

Supplementary Figure S2 Validation of overexpression and knockdown of ICP0-interacting proteins in Neuro-2a cells. **(A)** Neuro-2a cells were transfected with the indicated plasmids (400 ng/ml) for 48 h before being harvested for Western blot analysis for the indicated proteins. **(B)** Neuro-2a cells were transfected with the indicated siRNA (120 nM) for 48 h before being harvested for Western blot analysis. NC, negative control.

Supplementary Figure S3 Potentially ubiquitinated viral proteins as detected by mass spectrometry. Red color indicates proteins potentially targeted by ICP0 because their ubiquitination levels were increased in the presence relative to absence of ICP0 in both B/A and C/A comparisons (Figure 4).

Supplementary Table S1 Processed data for all mass spectrometry data used in this study. This table is provided as an attached Excel file.

Supplementary Table S2 Potential ICP0 substrates identified by ubiquitinome analysis classified by superfamilies according to conserved domains.

Supplementary Table S3 Primers used in this study.

## References

[B1] Alandijany T. Roberts A. P. E. Conn K. L. Loney C. Mcfarlane S. Orr A. (2018). Distinct temporal roles for the promyelocytic leukaemia (PML) protein in the sequential regulation of intracellular host immunity to HSV-1 infection. *PLoS Pathog.* 14:e1006769. 10.1371/journal.ppat.1006769 PMC5757968 29309427

[B2] Bendris N. Schmid S. L. (2017). Endocytosis, metastasis and beyond: multiple facets of SNX9. *Trends Cell Biol.* 27 189–200. 10.1016/j.tcb.2016.11.001 27989654 PMC5318277

[B3] Blum M. Chang H. Y. Chuguransky S. Grego T. Kandasaamy S. Mitchell A. (2020). The InterPro protein families and domains database: 20 years on. *Nucleic Acids Res*. 49 D344–D354. 10.1093/nar/gkaa977 33156333 PMC7778928

[B4] Boutell C. Everett R. D. (2003). The herpes simplex virus type 1 (HSV-1) regulatory protein ICP0 interacts with and Ubiquitinates p53. *J. Biol. Chem.* 278 36596–36602. 10.1074/jbc.M300776200 12855695

[B5] Boutell C. Canning M. Orr A. Everett R. D. (2005). Reciprocal activities between herpes simplex virus type 1 regulatory protein ICP0, a ubiquitin E3 ligase, and ubiquitin-specific protease USP7. *J. Virol.* 79 12342–12354. 10.1128/JVI.79.19.12342-12354.2005 16160161 PMC1211536

[B6] Boutell C. Cuchet-Lourenco D. Vanni E. Orr A. Glass M. Mcfarlane S. (2011). A viral ubiquitin ligase has substrate preferential SUMO targeted ubiquitin ligase activity that counteracts intrinsic antiviral defence. *PLoS Pathog.* 7:e1002245. 10.1371/journal.ppat.1002245 PMC3174244 21949651

[B7] Burleigh K. Maltbaek J. H. Cambier S. Green R. Gale M. Jr. James R. C. (2020). Human DNA-PK activates a STING-independent DNA sensing pathway. *Sci. Immunol.* 5:eaba4219. 10.1126/sciimmunol.aba4219 31980485 PMC7081723

[B8] Cai W. Z. Schaffer P. A. (1989). Herpes simplex virus type 1 ICP0 plays a critical role in the de novo synthesis of infectious virus following transfection of viral DNA. *J. Virol.* 63 4579–4589. 10.1128/JVI.63.11.4579-4589.1989 2552142 PMC251091

[B9] Cai W. Schaffer P. A. (1992). Herpes simplex virus type 1 ICP0 regulates expression of immediate-early, early, and late genes in productively infected cells. *J. Virol.* 66 2904–2915. 10.1128/JVI.66.5.2904-2915.1992 1313909 PMC241049

[B10] Canning M. Boutell C. Parkinson J. Everett R. D. (2004). A RING finger ubiquitin ligase is protected from autocatalyzed ubiquitination and degradation by binding to ubiquitin-specific protease USP7. *J. Biol. Chem.* 279 38160–38168. 10.1074/jbc.M402885200 15247261

[B11] Chaurushiya M. S. Lilley C. E. Aslanian A. Meisenhelder J. Scott D. C. Landry S. (2012). Viral E3 ubiquitin ligase-mediated degradation of a cellular E3: viral mimicry of a cellular phosphorylation mark targets the RNF8 FHA domain. *Mol. Cell* 46 79–90. 10.1016/j.molcel.2012.02.004 22405594 PMC3648639

[B12] Conwell S. E. White A. E. Harper J. W. Knipe D. M. (2015). Identification of TRIM27 as a novel degradation target of herpes simplex virus 1 ICP0. *J. Virol.* 89 220–229. 10.1128/JVI.02635-14 25320289 PMC4301158

[B13] Cuchet-Lourenco D. Anderson G. Sloan E. Orr A. Everett R. D. (2013). The viral ubiquitin ligase ICP0 is neither sufficient nor necessary for degradation of the cellular DNA sensor IFI16 during herpes simplex virus 1 infection. *J. Virol.* 87 13422–13432. 10.1128/JVI.02474-13 24089555 PMC3838218

[B14] Cuchet-Lourenco D. Vanni E. Glass M. Orr A. Everett R. D. (2012). Herpes simplex virus 1 ubiquitin ligase ICP0 interacts with PML isoform I and induces its SUMO-independent degradation. *J. Virol.* 86 11209–11222. 10.1128/JVI.01145-12 22875967 PMC3457127

[B15] Dembowski J. A. Deluca N. A. (2017). Purification of Viral DNA for the identification of associated viral and cellular proteins. *J. Vis. Exp*. 126:56374. 10.3791/56374 28892026 PMC5614390

[B16] dos Santos G. Rogel M. R. Baker M. A. Troken J. R. Urich D. Morales-Nebreda L. (2015). Vimentin regulates activation of the NLRP3 inflammasome. *Nat. Commun.* 6:6574. 10.1038/ncomms7574 25762200 PMC4358756

[B17] Drayman N. Karin O. Mayo A. Danon T. Shapira L. Rafael D. (2017). Dynamic proteomics of herpes simplex virus infection. *mBio* 8:e01612-17. 10.1128/mBio.01612-17 29114028 PMC5676043

[B18] Duarte L. F. Farias M. A. Alvarez D. M. Bueno S. M. Riedel C. A. Gonzalez P. A. (2019). Herpes simplex virus Type 1 infection of the central nervous system: insights into proposed interrelationships with neurodegenerative disorders. *Front. Cell. Neurosci.* 13:46. 10.3389/fncel.2019.00046 PMC6399123 30863282

[B19] Everett R. D. Boutell C. Orr A. (2004). Phenotype of a herpes simplex virus type 1 mutant that fails to express immediate-early regulatory protein ICP0. *J. Virol.* 78 1763–1774. 10.1128/jvi.78.4.1763-1774.2004 14747541 PMC369471

[B20] Everett R. D. Boutell C. Pheasant K. Cuchet-Lourenco D. Orr A. (2014). Sequences related to SUMO interaction motifs in herpes simplex virus 1 protein ICP0 act cooperatively to stimulate virus infection. *J. Virol.* 88 2763–2774. 10.1128/JVI.03417-13 24352468 PMC3958091

[B21] Everett R. D. Freemont P. Saitoh H. Dasso M. Orr A. Kathoria M. (1998). The disruption of ND10 during herpes simplex virus infection correlates with the Vmw110- and proteasome-dependent loss of several PML isoforms. *J. Virol.* 72 6581–6591. 10.1128/JVI.72.8.6581-6591.1998 9658103 PMC109835

[B22] Everett R. D. Meredith M. Orr A. Cross A. Kathoria M. Parkinson J. (1997). A novel ubiquitin-specific protease is dynamically associated with the PML nuclear domain and binds to a herpesvirus regulatory protein. *EMBO J* 16 566–577. 10.1093/emboj/16.3.566 9034339 PMC1169660

[B23] Everett R. D. Parsy M. L. Orr A. (2009). Analysis of the functions of herpes simplex virus type 1 regulatory protein ICP0 that are critical for lytic infection and derepression of quiescent viral genomes. *J. Virol.* 83 4963–4977. 10.1128/JVI.02593-08 19264778 PMC2682082

[B24] Fulzele A. Bennett E. J. (2018). Ubiquitin diGLY Proteomics as an approach to identify and quantify the ubiquitin-modified proteome. *Methods Mol. Biol.* 1844 363–384. 10.1007/978-1-4939-8706-1_23 30242721 PMC6791129

[B25] Gu H. (2016). Infected cell protein 0 functional domains and their coordination in herpes simplex virus replication. *World J. Virol.* 5 1–13. 10.5501/wjv.v5.i1.1 26870669 PMC4735549

[B26] Gu H. Liang Y. Mandel G. Roizman B. (2005). Components of the REST/CoREST/histone deacetylase repressor complex are disrupted, modified, and translocated in HSV-1-infected cells. *Proc. Natl. Acad. Sci. U.S.A.* 102 7571–7576. 10.1073/pnas.0502658102 15897453 PMC1140450

[B27] Halford W. P. Schaffer P. A. (2001). ICP0 is required for efficient reactivation of herpes simplex virus type 1 from neuronal latency. *J. Virol.* 75 3240–3249. 10.1128/JVI.75.7.3240-3249.2001 11238850 PMC114117

[B28] Halford W. P. Kemp C. D. Isler J. A. Davido D. J. Schaffer P. A. (2001). ICP0, ICP4, or VP16 expressed from adenovirus vectors induces reactivation of latent herpes simplex virus type 1 in primary cultures of latently infected trigeminal ganglion cells. *J. Virol.* 75 6143–6153. 10.1128/JVI.75.13.6143-6153.2001 11390616 PMC114330

[B29] Hendriks I. A. Lyon D. Young C. Jensen L. J. Vertegaal A. C. Nielsen M. L. (2017). Site-specific mapping of the human SUMO proteome reveals co-modification with phosphorylation. *Nat. Struct. Mol. Biol.* 24 325–336. 10.1038/nsmb.3366 28112733

[B30] Jan Fada B. Kaadi E. Samrat S. K. Zheng Y. Gu H. (2020). Effect of SUMO-SIM interaction on the ICP0-mediated degradation of PML isoform II and its associated proteins in herpes simplex virus 1 infection. *J. Virol.* 94:e00470-20. 10.1128/JVI.00470-20 32295906 PMC7307090

[B31] Jassal B. Matthews L. Viteri G. Gong C. Lorente P. Fabregat A. (2020). The reactome pathway knowledgebase. *Nucleic Acids Res.* 48 D498–D503. 31691815 10.1093/nar/gkz1031 PMC7145712

[B32] Jurak I. Silverstein L. B. Sharma M. Coen D. M. (2012). Herpes simplex virus is equipped with RNA- and protein-based mechanisms to repress expression of ATRX, an effector of intrinsic immunity. *J. Virol.* 86 10093–10102. 10.1128/JVI.00930-12 22787211 PMC3446562

[B33] Kalamvoki M. Qu J. Roizman B. (2008). Translocation and colocalization of ICP4 and ICP0 in cells infected with herpes simplex virus 1 mutants lacking glycoprotein E, glycoprotein I, or the virion host shutoff product of the UL41 gene. *J. Virol.* 82 1701–1713. 10.1128/JVI.02157-07 18057247 PMC2258734

[B34] Katzenell S. Cabrera J. R. North B. J. Leib D. A. (2017). Isolation, purification, and culture of primary murine sensory neurons. *Methods Mol. Biol.* 1656 229–251. 10.1007/978-1-4939-7237-1_15 28808974 PMC5613752

[B35] Kim E. T. Dybas J. M. Kulej K. Reyes E. D. Price A. M. Akhtar L. N. (2021). Comparative proteomics identifies Schlafen 5 (SLFN5) as a herpes simplex virus restriction factor that suppresses viral transcription. *Nat. Microbiol*. 6 234–245. 10.1038/s41564-020-00826-3 33432153 PMC7856100

[B36] Knipe D. M. Howley P. M. (2013). *Fields Virology.* Philadelphia, PA: Wolters Kluwer.

[B37] Lanfranca M. P. Mostafa H. H. Davido D. J. (2014). HSV-1 ICP0: an E3 ubiquitin ligase that counteracts host intrinsic and innate immunity. *Cells* 3 438–454. 10.3390/cells3020438 24852129 PMC4092860

[B38] Lilley C. E. Chaurushiya M. S. Boutell C. Landry S. Suh J. Panier S. (2010). A viral E3 ligase targets RNF8 and RNF168 to control histone ubiquitination and DNA damage responses. *EMBO J.* 29 943–955. 10.1038/emboj.2009.400 20075863 PMC2837166

[B39] Liu X. Ma Y. Voss K. Van Gent M. Chan Y. K. Gack M. U. (2021). The herpesvirus accessory protein gamma134.5 facilitates viral replication by disabling mitochondrial translocation of RIG-I. *PLoS Pathog.* 17:e1009446. 10.1371/journal.ppat.1009446 PMC7996975 33770145

[B40] Liuyu T. Yu K. Ye L. Zhang Z. Zhang M. Ren Y. (2019). Induction of OTUD4 by viral infection promotes antiviral responses through deubiquitinating and stabilizing MAVS. *Cell Res.* 29 67–79. 10.1038/s41422-018-0107-6 30410068 PMC6318273

[B41] Mallon S. Wakim B. T. Roizman B. (2012). Use of biotinylated plasmid DNA as a surrogate for HSV DNA to identify proteins that repress or activate viral gene expression. *Proc. Natl. Acad. Sci. U.S.A.* 109 E3549–E3557. 10.1073/pnas.1218783109 23223531 PMC3529035

[B42] Mevissen T. E. Hospenthal M. K. Geurink P. P. Elliott P. R. Akutsu M. Arnaudo N. (2013). OTU deubiquitinases reveal mechanisms of linkage specificity and enable ubiquitin chain restriction analysis. *Cell* 154 169–184. 10.1016/j.cell.2013.05.046 23827681 PMC3705208

[B43] Orzalli M. H. Broekema N. M. Diner B. A. Hancks D. C. Elde N. C. Cristea I. M. (2015). cGAS-mediated stabilization of IFI16 promotes innate signaling during herpes simplex virus infection. *Proc. Natl. Acad. Sci. U.S.A.* 112 E1773–E1781. 10.1073/pnas.1424637112 25831530 PMC4394261

[B44] Orzalli M. H. Deluca N. A. Knipe D. M. (2012). Nuclear IFI16 induction of IRF-3 signaling during herpesviral infection and degradation of IFI16 by the viral ICP0 protein. *Proc. Natl. Acad. Sci. U.S.A.* 109 E3008–E3017. 10.1073/pnas.1211302109 23027953 PMC3497734

[B45] Pan D. Coen D. M. (2012). Quantification and analysis of thymidine kinase expression from acyclovir-resistant G-string insertion and deletion mutants in herpes simplex virus-infected cells. *J. Virol.* 86 4518–4526. 10.1128/JVI.06995-11 22301158 PMC3318614

[B46] Pan D. Flores O. Umbach J. L. Pesola J. M. Bentley P. Rosato P. C. (2014). A neuron-specific host microRNA targets herpes simplex virus-1 ICP0 expression and promotes latency. *Cell Host Microbe* 15 446–456. 10.1016/j.chom.2014.03.004 24721573 PMC4142646

[B47] Pan D. Li G. Morris-Love J. Qi S. Feng L. Mertens M. E. (2019). Herpes simplex virus 1 lytic infection blocks MicroRNA (miRNA) biogenesis at the stage of nuclear export of pre-miRNAs. *mBio* 10:e02856-18. 10.1128/mBio.02856-18 30755517 PMC6372804

[B48] Parkinson J. Lees-Miller S. P. Everett R. D. (1999). Herpes simplex virus type 1 immediate-early protein vmw110 induces the proteasome-dependent degradation of the catalytic subunit of DNA-dependent protein kinase. *J Virol* 73 650–657. 10.1128/JVI.73.1.650-657.1999 9847370 PMC103871

[B49] Raja P. Lee J. S. Pan D. Pesola J. M. Coen D. M. Knipe D. M. (2016). A herpesviral lytic protein regulates the structure of latent viral chromatin. *mBio* 7:e00633-16. 10.1128/mBio.00633-16 27190217 PMC4895110

[B50] Ramos I. Stamatakis K. Oeste C. L. Perez-Sala D. (2020). Vimentin as a multifaceted player and potential therapeutic target in viral infections. *Int. J. Mol. Sci.* 21:4675. 10.3390/ijms21134675 32630064 PMC7370124

[B51] Rodriguez M. C. Dybas J. M. Hughes J. Weitzman M. D. Boutell C. (2020). The HSV-1 ubiquitin ligase ICP0: modifying the cellular proteome to promote infection. *Virus Res.* 285:198015. 10.1016/j.virusres.2020.198015 32416261 PMC7303953

[B52] Rose C. M. Isasa M. Ordureau A. Prado M. A. Beausoleil S. A. Jedrychowski M. P. (2016). Highly multiplexed quantitative mass spectrometry analysis of ubiquitylomes. *Cell Syst.* 3 395–403.e4. 10.1016/j.cels.2016.08.009 27667366 PMC5241079

[B53] Sandri-Goldin R. M. Sekulovich R. E. Leary K. (1987). The alpha protein ICP0 does not appear to play a major role in the regulation of herpes simplex virus gene expression during infection in tissue culture. *Nucleic Acids Res.* 15 905–919. 10.1093/nar/15.3.905 3029709 PMC340497

[B54] Sato Y. Kato A. Maruzuru Y. Oyama M. Kozuka-Hata H. Arii J. (2016b). Cellular transcriptional coactivator RanBP10 and herpes simplex virus 1 ICP0 interact and synergistically promote viral gene expression and replication. *J. Virol.* 90 3173–3186. 10.1128/JVI.03043-15 26739050 PMC4810668

[B55] Sato Y. Kato A. Arii J. Koyanagi N. Kozuka-Hata H. Oyama M. (2016a). Ubiquitin-specific protease 9X in host cells interacts with herpes simplex virus 1 ICP0. *J. Vet. Med. Sci.* 78 405–410. 10.1292/jvms.15-0598 26596467 PMC4829507

[B56] Sloan E. Orr A. Everett R. D. (2016). MORC3, a component of PML nuclear bodies, has a role in restricting herpes simplex virus 1 and human cytomegalovirus. *J. Virol.* 90 8621–8633. 10.1128/JVI.00621-16 27440897 PMC5021396

[B57] Sloan E. Tatham M. H. Groslambert M. Glass M. Orr A. Hay R. T. (2015). Analysis of the SUMO2 Proteome during HSV-1 Infection. *PLoS Pathog.* 11:e1005059. 10.1371/journal.ppat.1005059 PMC4511656 26200910

[B58] Sun B. Yang X. Hou F. Yu X. Wang Q. Oh H. (2021). Regulation of host and virus genes by neuronal miR-138 favours herpes simplex virus 1 latency. *Nat. Microbiol*. 6 682–696. 10.1038/s41564-020-00860-1 33558653 PMC8221016

[B59] Sun Z. Li W. Xu J. Ren K. Gao F. Jiang Z. (2020). Proteomic analysis of cerebrospinal fluid in children with acute enterovirus-associated meningoencephalitis identifies dysregulated host processes and potential biomarkers. *J. Proteome Res.* 19 3487–3498. 10.1021/acs.jproteome.0c00307 32678604

[B60] Szklarczyk D. Gable A. L. Lyon D. Junge A. Wyder S. Huerta-Cepas J. (2019). STRING v11: protein-protein association networks with increased coverage, supporting functional discovery in genome-wide experimental datasets. *Nucleic Acids Res.* 47 D607–D613. 10.1093/nar/gky1131 30476243 PMC6323986

[B61] Taylor K. E. Mossman K. L. (2015). Cellular protein WDR11 interacts with specific herpes simplex virus proteins at the trans-golgi network to promote virus replication. *J. Virol.* 89 9841–9852. 10.1128/JVI.01705-15 26178983 PMC4577907

[B62] Thompson R. L. Sawtell N. M. (2006). Evidence that the herpes simplex virus type 1 ICP0 protein does not initiate reactivation from latency in vivo. *J. Virol.* 80 10919–10930. 10.1128/JVI.01253-06 16943285 PMC1642178

[B63] Tischer B. K. Von Einem J. Kaufer B. Osterrieder N. (2006). Two-step red-mediated recombination for versatile high-efficiency markerless DNA manipulation in *Escherichia coli*. *Biotechniques* 40 191–197. 10.2144/000112096 16526409

[B64] Udeshi N. D. Mani D. C. Satpathy S. Fereshetian S. Gasser J. A. Svinkina T. (2020). Rapid and deep-scale ubiquitylation profiling for biology and translational research. *Nat. Commun.* 11:359. 10.1038/s41467-019-14175-1 31953384 PMC6969155

[B65] Udeshi N. D. Mertins P. Svinkina T. Carr S. A. (2013). Large-scale identification of ubiquitination sites by mass spectrometry. *Nat. Protoc.* 8 1950–1960. 10.1038/nprot.2013.120 24051958 PMC4725055

[B66] Vanni E. Gatherer D. Tong L. Everett R. D. Boutell C. (2012). Functional characterization of residues required for the herpes simplex virus 1 E3 ubiquitin ligase ICP0 to interact with the cellular E2 ubiquitin-conjugating enzyme UBE2D1 (UbcH5a). *J. Virol.* 86 6323–6333. 10.1128/JVI.07210-11 22438555 PMC3372195

[B67] Waisner H. Kalamvoki M. (2019). The ICP0 protein of herpes simplex virus 1 (HSV-1) downregulates major autophagy adaptor proteins sequestosome 1 and optineurin during the early stages of HSV-1 infection. *J. Virol.* 93:e01258-19. 10.1128/JVI.01258-19 31375597 PMC6803258

[B68] Wilcox C. L. Smith R. L. Everett R. D. Mysofski D. (1997). The herpes simplex virus type 1 immediate-early protein ICP0 is necessary for the efficient establishment of latent infection. *J. Virol.* 71 6777–6785. 10.1128/JVI.71.9.6777-6785.1997 9261402 PMC191958

[B69] Xie C. Mao X. Huang J. Ding Y. Wu J. Dong S. (2011). KOBAS 2.0: a web server for annotation and identification of enriched pathways and diseases. *Nucleic Acids Res.* 39 W316–W322. 10.1093/nar/gkr483 21715386 PMC3125809

[B70] Yang B. Jia Y. Meng Y. Xue Y. Liu K. Li Y. (2022). SNX27 suppresses SARS-CoV-2 infection by inhibiting viral lysosome/late endosome entry. *Proc. Natl. Acad. Sci. U.S.A.* 119:e2117576119. 10.1073/pnas.2117576119 35022217 PMC8794821

[B71] Yao F. Schaffer P. A. (1994). Physical interaction between the herpes simplex virus type 1 immediate-early regulatory proteins ICP0 and ICP4. *J. Virol.* 68 8158–8168. 10.1128/JVI.68.12.8158-8168.1994 7966607 PMC237281

[B72] Zhang J. Wang K. Wang S. Zheng C. (2013). Herpes simplex virus 1 E3 ubiquitin ligase ICP0 protein inhibits tumor necrosis factor alpha-induced NF-kappaB activation by interacting with p65/RelA and p50/NF-kappaB1. *J. Virol.* 87 12935–12948. 10.1128/JVI.01952-13 24067962 PMC3838126

[B73] Zhao Y. Majid M. C. Soll J. M. Brickner J. R. Dango S. Mosammaparast N. (2015). Noncanonical regulation of alkylation damage resistance by the OTUD4 deubiquitinase. *EMBO J.* 34 1687–1703. 10.15252/embj.201490497 25944111 PMC4475402

[B74] Zheng Y. Samrat S. K. Gu H. (2016). A tale of two PMLs: elements regulating a differential substrate recognition by the ICP0 E3 ubiquitin ligase of herpes simplex virus 1. *J. Virol.* 90 10875–10885. 10.1128/JVI.01636-16 27681131 PMC5110152

