# Supplementary material for: Interactome and Ubiquitinome Analyses Identify Functional Targets of Herpes Simplex Virus 1 Infected Cell Protein 0
Source: Front Microbiol. 2022 Apr 18;13:856471. doi: 10.3389/fmicb.2022.856471 (PMC9062659; doi:10.3389/fmicb.2022.856471)
Supplement: Supplementary Figure S1 — ICP0-interacting network. The functional clusters were generated by the K-mean approach based on the candidate ICP0-interacting proteins identified by the interactome analysis in all three cell lines. [file Data_Sheet_1.docx]

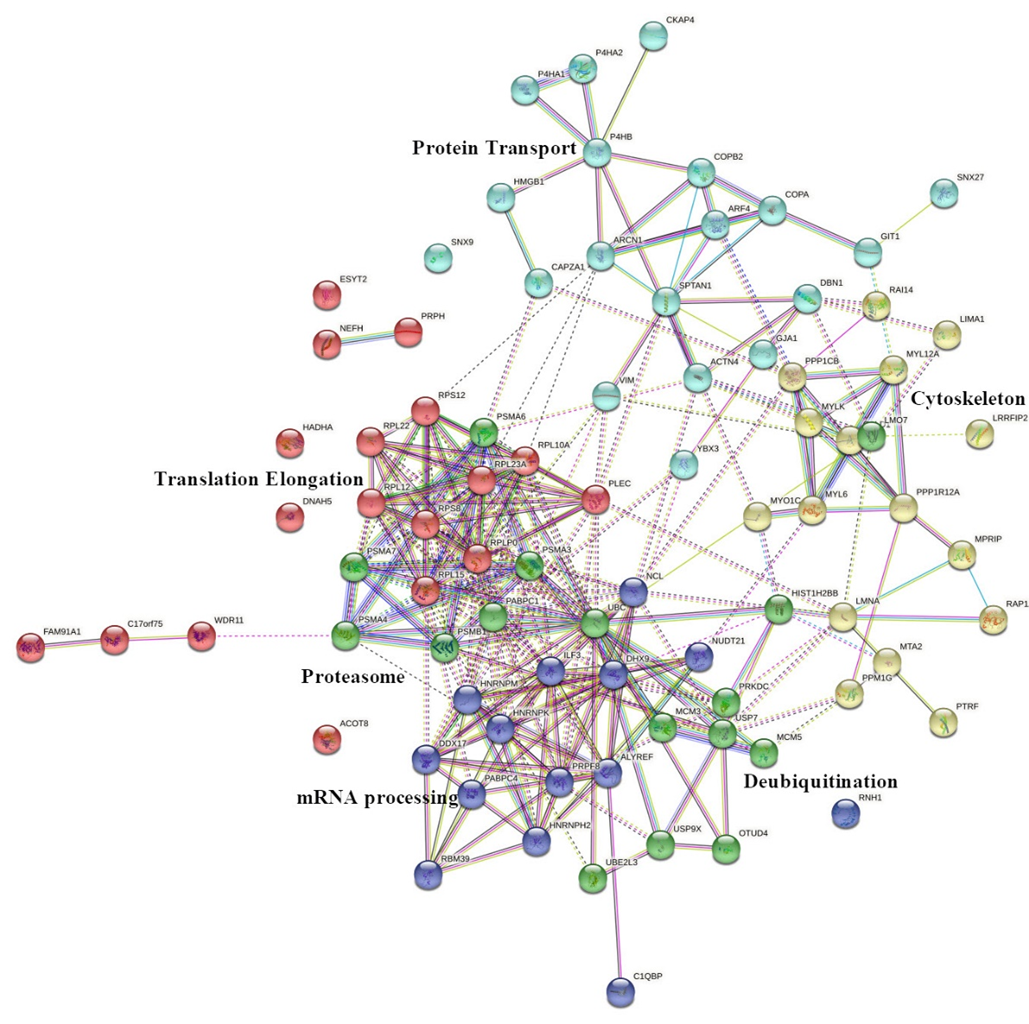


**Fig. S1. ICP0-interacting network.** The functional clusters were generated by the K-mean approach based on the candidate ICP0-interacting proteins identified by the interactome analysis in all three cell lines.

**
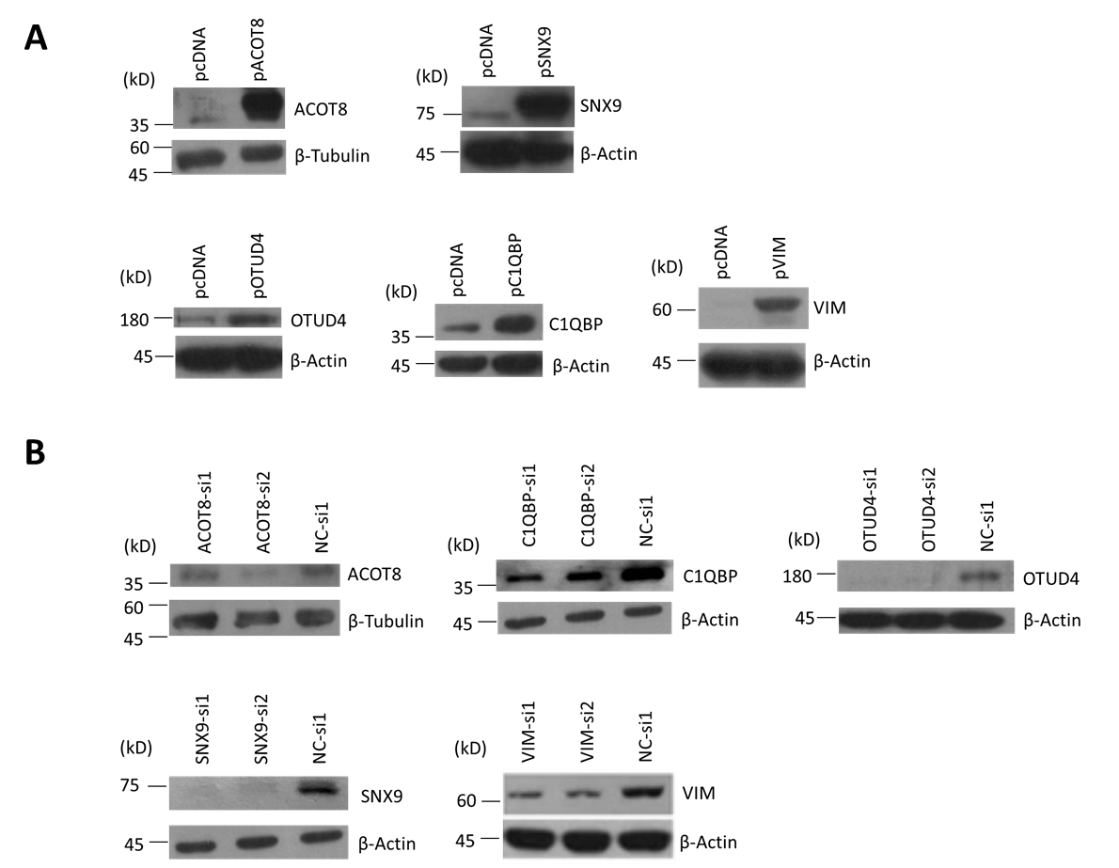
**

**Fig. S2. Validation of overexpression and knockdown of ICP0-interacting proteins in Neuro-2a cells.** (A) Neuro-2a cells were transfected with 400 ng/ml of the indicated plasmids for 48 h before being harvested for western blot analysis for the indicated proteins. (B) Neuro-2a cells were transfected with 120 nM of the indicated siRNA for 48 h before being harvested for western blot analysis. NC, negative control.


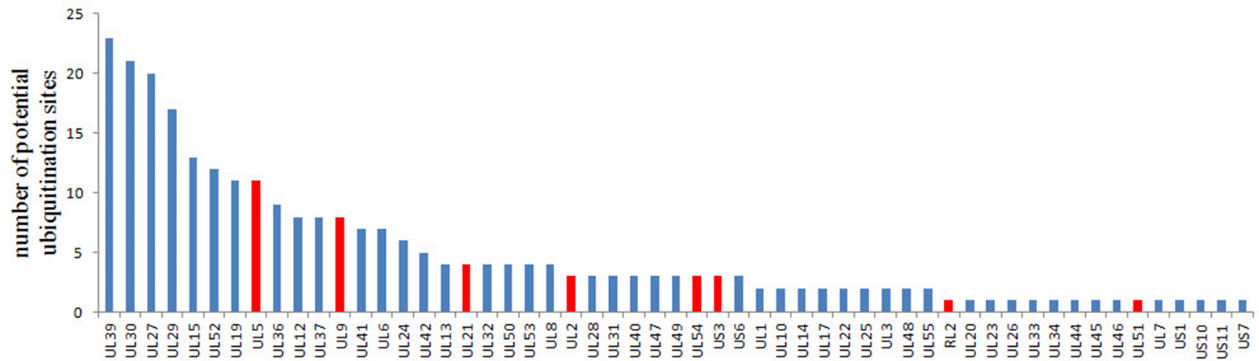


**Fig. S3. Potentially ubiquitinated viral proteins as detected by mass spectrometry.** Red color indicates proteins potentially targeted by ICP0 because their ubiquitination levels were increased in the presence relative to absence of ICP0 in both B/A and C/A comparisons (Fig. 4).

**Table S1. Processed data for all mass spectrometry data used in this study. This table is provided as an attached Excel file.**

**Table S2. Potential ICP0 substrates identified by ubiquitinome analysis classified by superfamilies according to conserved domains**

| Term description | Matching proteins |
| --- | --- |
| P-loop containing nucleoside triphosphate hydrolase | DDX3X, MPP2, DLG4, SMC6, GTPBP4, PSMC1, WRNIP1, PSMC6, HELLS, EIF4A3, NVL, ORC4, VCP, GNL2, ABCF2, DDX54, WRN, SRPR, ASCC3, HNRNPUL1, DHX9, EEF2, KIF2C, ATL2, DDX10, DDX1, ERCC6, UPF1, SMC5, CHD8, RNF213, DDX24, GNL3L, KIF3A, LSG1, CHD2, BLM, KATNA1, KIF20A |
| Zinc finger, RING/FYVE/PHD-type | UHRF1, RUFY1, UHRF2, HGS, RNF34, LTN1, BAZ1A, USP5, TRAFD1, RNF149, WHSC1, ZMYND11, PML, RNF213, TTC3, USP33, RNF168, BAZ2A |
| Nucleotide-binding alpha-beta plait domain superfamily | ELAVL3, RBM28, G3BP1, NCBP3, NIFK, RBM17, RALY, UPF3B, U2SURP, PABPC4, TARDBP, RBM27, HNRNPC, CELF4, SRSF6, PTBP1, PARP10, HNRNPF |
| Armadillo-type fold | IFRD1, UTP20, CTNNB1, CEBPZ, HUWE1, PSMD1, PSMD5, SDAD1, PI4KA, LTN1, THADA, MYBBP1A, BZW1, EIF4G3, CLTC, XPO1, RALGAPA1, PUM2 |
| RNA-binding domain | ELAVL3, RBM28, G3BP1, NIFK, RBM17, RALY, UPF3B, U2SURP, PABPC4, TARDBP, RBM27, HNRNPC, CELF4, SRSF6, PTBP1, HNRNPF |
| DEAD/DEAH box helicase domain | DDX3X, EIF4A3, DDX54, WRN, ASCC3, DHX9, DDX10, DDX1, DDX24, BLM |
| Ubiquitin interacting motif | ATXN3, HGS, STAM, UIMC1 |
| Ubiquitin specific protease domain | USP15, USP43, USP11, USP5, USP33, USP7 |

**Table S3. Primers used in this study**

| Gene names | Primer names | | Sequences (5’ – 3’) |
| --- | --- | --- | --- |
| BAC construction |  |  | |
| *Flag-ICP0* | ICP0Kan-F | ACCCTCCGTCAGCGACCCTCCAGCCGCATACGACCCCCATGGACTACAAGGATGACGACGATAAGGAGCCCCGCCTAGGGATAACAGGGTAATCGATTT | |
|  | ICP0Kan-R | CGGCCCTCAGGCCGGCGGGTACTCGCTCCGGGGCGGGGCTCCTTATCGTCGTCATCCTTGTAGTCCATGGGGGTGCCAGTGTTACAACCAATTAACC | |
|  | ICP0Zeo-F | ACCCTCCGTCAGCGACCCTCCAGCCGCATACGACCCCCATGGACTACAAGGATGACGACGATAAGGAATTCTGTTGACAATTAATCATCGGCAT | |
|  | ICP0Zeo-R | CGGCCCTCAGGCCGGCGGGTACTCGCTCCGGGGCGGGGCTCCTTATCGTCGTCATCCTTGTAGTCTCAGTCCTGCTCCTCGGCCA | |
| plasmid construction |  |  | |
| *VIM* | VIM-F | CGGAATTCATGTCCACCAGGTCCGTGTC | |
|  | VIM-R | CGGAATTCATGTCCACCAGGTCCGTGTC | |
| *HBB-BS* | HBB-BS-F | CCGCTCGAGATGGTGCACCTGACTGATGCTG | |
|  | HBB-BS-R | CGGAATTCTTAGTGGTACTTGTGAGCCAGGGCA | |
| *UL50* | UL50-F | CGGAATTCATGAGTCAGTGGGGATCCGGG | |
|  | UL50-R | CCAAGCTTCTAAATACCGGTAGAGCCAAAACCC | |
| *UBE2L3* | UBE2L3-F | CGGAATTCATGGCGGCCAGCAGGAG | |
|  | UBE2L3-R | CCAAGCTTTTAGTCCACAGGTCGCTTTTCC | |
| *PSMA3* | PSMA3-F | CGGAATTCATGAGCTCCATCGGCACTGG | |
|  | PSMA3-R | CGGGATCCTTACATATTGTCATCATCTGATTCA | |
| *HNRNPM* | HNRNPM-F | GCTCTAGAATGGCGGCAGGGGTCGAA | |
|  | HNRNPM-R | GGGGTACCTTAAGCGTTTCTATCAATTCGAACG | |
| *DDX17* | DDX17-F | CGGAATTCCTGCCCACCGGCTTTGTAGC | |
|  | DDX17-R | CCAAGCTTTCATTTACGTGAAGGAGGAGGAGGG | |
| *C1QBP* | C1QBP-F | CCGCTCGAGATGCTGCCTCTGCTGCGCT | |
|  | C1QBP-R | CGGGATCCCTACTGGCTCTTGACAAAACTCTTGAGG | |
| *Hist1H2BB* | Hist1H2BB-F | CCGCTCGAGATGCCTGAACCCTCTAAGTCTGC | |
|  | Hist1H2BB-R | CGGGATCCTTATTTAGAGCTAGTGTACTTGGTAACTGC | |
| *DBN1* | DBN1-F | CCGCTCGAGAAGAAGTCAGAGTCGGAGGTGGA | |
|  | DBN1-R | CGGGATCCCTAATCACCACCCTCGAAGCC | |
| *PSMA6* | PSMA6-F | CCGCTCGAGATGTCCCGTGGTTCCAGCG | |
|  | PSMA6-R | CGGGATCCTTAGTCTCTCTCTGCTAGAGCAACAAGG | |
| *P4HA2* | P4HA2-F | CCGCTCGAGATGAAACTCTGGGTGTCTGCATT | |
|  | P4HA2-R | CGGGATCCTCAGTCAACTTCTGTTGATCCACAAGGTCTC | |
| *MCM3* | MCM3-F | CGGAATTCATGGCGGGTACCGTGGTGC | |
|  | MCM3-R | CCAAGCTTTCAACTGAAGAGAAGGGCTCTTCG | |
| *MCM5* | MCM5-F | CGGAATTCATGTCGGGATTCGACGATC | |
|  | MCM5-R | CCAAGCTTTCACTTGAGGCGGTAGAGAAC | |
| *RAP1A* | RAP1A-F | CCGCTCGAGATGCGTGAGTACAAGCTAGTGGTCC | |
|  | RAP1A-R | CGGAATTCGAGCAGCAGACATGATTTCTTTTTA | |
| *OTUD4* | OTUD4-F | CCGCTCGAGATGGCCTGTATTCACTATCTTCGAG | |
|  | OTUD4-R | CGGGATCCTCAAGTGTGCTGTCCCCTATGG | |
| *SNX9* | SNX9-F | CCGCTCGAGATGGCCACCAAGGCTCGGGT | |
|  | SNX9-R | CGGGATCCCTACATCACTGGAAAGCGGCTGAG | |
| *ACOT8* | ACOT8-F | CGGAATTCATGTCGTCCCCGCAGG | |
|  | ACOT8-R | CCAAGCTTCTACAGCTTGCTCTCTGAGACCTGG | |
| *RPL10A* | RPL10A-F | CCGCTCGAGATGAGCAGCAAAGTCTCTCGCGAC | |
|  | RPL10A-R | CCCAAGCTTTTAATATAGGCGCTGGGGCTT | |
| *MYL12A* | MYL12A-F | CCGCTCGAGATGTCGAGCAAAAGAACAAAGAC | |
|  | MYL12A-R | CCCAAGCTTTCAGTCATCTTTGTCTTTGGCTC | |
| *PPP1CB* | PPP1CB-F | CCGCTCGAGGCGGACGGGGAGCTGAAC | |
|  | PPP1CB-R | CCCAAGCTTTCACCTTTTCTTCGGCGGAT | |
| *CAVIN1* | CAVIN1-F | CCGCTCGAGATGGAGGACCCCACGCTCTATATT | |
|  | CAVIN1-R | CCCAAGCTTTCAGTCGCTGTCGCTCTTGT | |
| *Ubiquitin* | Ubiquitin-F | CGGGATCCGATGCAGATCTTCGTGAAAACCCTT | |
|  | Ubiquitin-R | CCAAGCTTTTAACAGCCACCCCTCAGGC | |
